# Screening and Conjoint Analysis of Key lncRNAs for Milk Fat Metabolism in Dairy Cows

**DOI:** 10.3389/fgene.2022.772115

**Published:** 2022-02-03

**Authors:** Tong Mu, Honghong Hu, Xiaofang Feng, Yanfen Ma, Ying Wang, Jiamin Liu, Baojun Yu, Wan Wen, Juan Zhang, Yaling Gu

**Affiliations:** ^1^ School of Agriculture, Ningxia University, Yinchuan, China; ^2^ Key Laboratory of Ruminant Molecular and Cellular Breeding, Ningxia Hui Autonomous Region, Ningxia University, Yinchuan, China; ^3^ Animal Husbandry Extension Station, Yinchuan, China

**Keywords:** Holstein cattle, lncRNAs, gene, conjoint analysis, milk fat percentage

## Abstract

Long noncoding RNAs (lncRNAs) play an important regulatory role in various biological processes as a key regulatory factor. However, the complete expression profile of lncRNAs in dairy cows and its function in milk fat synthesis are unknown. In this study, RNA sequencing (RNA-seq) was used to research the whole genome expression of lncRNAs and mRNA transcripts in high and low milk fat percentage (MFP) bovine mammary epithelial cells (BMECs), and joint analysis was carried out. We identified a total of 47 differentially expressed genes (DEGs) and 38 differentially expressed lncRNAs (DELs, Padj <0.05), enrichment analysis screened out 11 candidate DEGs that may regulate milk fat metabolism. Downregulated differential gene *ENPP2* (The expression level in BMECs of high milk fat dairy cows was lower than that of low milk fat cows) and upregulated differential gene *BCAT1* are more likely to participate in the milk fat metabolism, and its function needs further experiments verification. The enrichment analysis of target genes predicted by DELs identified 7 cis (co-localization) and 10 trans (co-expression) candidate target genes related to milk lipid metabolism, corresponding to a total of 18 DELs. Among them, the targeting relationship between long intervening/intergenic noncoding RNA (lincRNA) TCONS_00082721 and *FABP4* is worthy of attention. One hundred and fifty-six competing endogenous RNAs (ceRNAs) interaction regulation networks related to milk fat metabolism were constructed based on the expression information of DELs, differential microRNAs (miRNAs), and lipid metabolism-related target genes. The regulatory network centered on miR-145 will be the focus of subsequent experimental research. The ceRNAs regulatory network related to TCONS_00082721 and TCONS_00172817 are more likely to be involved in milk fat synthesis. These results will provide new ways to understand the complex biology of dairy cow milk fat synthesis and provide valuable information for breed improvement of Chinese Holstein cow.

## Introduction

Milk quality is affected by many factors, such as population genetic structure, reproductive performance, feeding and management, and is closely related to the main raw material composition of milk. Milk fat is an important component in butter and yogurt, and its content and composition are the main reference elements in milk quality evaluation (Mu et al., 2021). Nowadays, milk fat content is not only one of the important indicators of core competitiveness of dairy industry, but also the main target feature of dairy cow breeding (Li et al., 2015). To a certain extent, it also plays an important role in nutrition and metabolism during human growth and development (Chen et al., 2019). Therefore, theory exploration on milk fat formation and regulation and the improvement of milk fat content become one the focuses of lactating biology research.

In the past, many scholars have extensively studied the complex regulatory mechanisms of mammary gland development and elucidated the main pathways of milk fat synthesis (including *de novo* synthesis and absorption of FA in the blood) (Bauman et al., 2006). Quantitative real-time reverse transcription PCR (qRT-PCR) can better discover the transcriptional regulation steps of milk fat synthesis. High-throughput sequencing has identified a series of effective genes and regulatory factors in milk fat metabolism (Xu et al., 2010). The study of gene regulation mainly depends on protein-coding genes. However, more and more evidence suggests that non-coding RNAs (ncRNAs), which are mistaken for “transcriptional noise”, are also important factors in regulating complex developmental processes in organisms (Ma et al., 2017). Long noncoding RNAs (lncRNAs) are newly identified long-stranded non-coding RNAs in the breast. It is more than 200 nucleotides in length and is more tissue-specific than coding genes (Xu et al., 2016). lncRNAs have a more complex mode of action on genes than microRNAs (miRNAs). Currently, transcriptional interference is a more mature regulatory model of regulation (Fatica and Bozzoni, 2014). Functional studies have found that lncRNAs regulate gene expression at epigenetic, transcriptional and post-transcriptional levels in mammals, and also play key regulatory roles in a variety of biological processes (Yeong-Hwan et al., 2018). In regulating milk fat metabolism in goats, Yu et al. (2017) found that compared with early lactation, the expression trend of lncRNAs TCONS-00144434, TCONS-00148514 and TCONS-00055666 during mature lactation was the same as that of *FASN*, *LPL*, *GPAM* and *MSMO1* with the function of fatty acid biosynthesis and cholesterol storage (the full names of all genes in this paper are shown in Supplementary Table S1). However, the specific regulatory mechanism needs to be further studied. Chao et al. (2017) found that the target genes of TCONS_00162862 were involved in lipid transporter protein activity, ligase activity, and fatty acid transport in dairy cow, and mainly enriched in MAPK, PI3K-Akt, and insulin signaling pathways. Zheng et al. (2018) performed transcriptome sequencing on four breast tissues of Holstein cows during peak lactation and late lactation, and found that the target genes of differentially expressed LNC-XLOC_274111 were *FABP3* and *FABP4*, which would encode fatty acid-binding proteins in the bovine mammary gland. The target genes of XLOC_000752 were significantly enriched in PPAR, AMPK, and insulin signaling pathways, as well as glycerol metabolism pathways.

So far, research on lncRNAs in milk fat metabolism of ruminants are scarce. The functions and expression profiles of lncRNAs in the bovine mammary gland are still unclear, and there are even fewer studies on differentially expressed lncRNAs (DELs) for differences in high and low milk fat percentage (MFP). In addition, the dynamic pattern of the interaction of ceRNA in milk fat synthesis remains is unknown. To better understand the metabolic process of milk fat synthesis during lactation, Holstein cows with extreme differences in MFP were screened, fresh milk was collected and bovine mammary epithelial cells (BMECs) were isolated. The differentially expressed genes (DEGs) and DELs related to milk fat synthesis in the same lactation stage were screened by transcriptomics technology, and the predicting lncRNAs to target miRNAs and constructing a ceRNA regulatory network of genes, miRNAs and lncRNAs related to milk fat metabolism. It is an important step in understanding the complex biological processes involved in milk production, and provides valuable information for the improvement of Chinese Holstein cow breeds.

## Materials and Methods

### Ethics Statement

In this study, BMECs were isolated and cultured from milk samples of dairy cows. Two hundred milliliter milk samples were collected from each dairy cow, and aseptic operation was strictly carried out during the sampling process, so there was no harm to dairy cows.

### Experimental Cow Screening and Milk Sample Collection

245 first calving Holstein cows with the same feeding background (Table 1), similar age and in the middle and late stage of lactation (150–220 days) were selected from Ningxia Nongkeng Helanshan Maosheng dairy farm. Early, middle and late milk samples of each cow were collected for dairy herd improvement (DHI) determination. Holstein cows with high MFP (*n* = 4) and low MFP (*n* = 4) with somatic cell count (SCC) less than 100,000/mL were selected (Table 2). Fresh milk samples were aseptically collected into 50 ml centrifuge tubes, and four tubes per cow were collected for a total of 200 ml. Tighten the cap of the bottle, seal and sterilize it, put it in a thermos flask containing 37°C sterile water, and return it to the laboratory for later use.

**TABLE 1:** Ingredient and nutrient composition in diet (dry matter basis) %.

Ingredient	Content	Composition	Content
Alfalfa	16.22	Dry matter (kg)	17.28
Corn silage	51.32	Net energy for lactating cow (MJ/kg)	7.76
Tablet corn	10.82	Crude protein	18.31
Soybean meal	10.82	Neutral detergent fiber	35.84
Cotton meal	5.41	Acid detergent fiber	21.85
10 % premix	5.41	Fat	2.59
		Ca	0.52
Total	100	P	0.35

Note: Each kg of premix contains 800,000 IU, of V_A_, 200,000 IU, of V_D_, 4,000 mg of V_E_, 1,200 mg of Cu, 6,000 mg of Fe, 4,000 mg of Mn, 4,000 mg of Zn, 40 mg of I, 40 mg of Co and 32 mg of Se.

**TABLE 2:** High and low MFP and SCC of Holstein cow.

Items	Number	Age	Lactation days	MFP (%)	SCC (10,000/ml)
High-milk fat group	H_2098	30	186	4.82	5
	H_2046	31	189	4.54	2
	H_2226	29	160	4.74	9
	H_2190	29	157	4.88	5
Low-milk fat group	L_2034	31	187	2.60	6
	L_2037	31	175	2.81	5
	L_2170	30	207	2.85	8
	L_2137	29	150	2.84	7

Number: number of each cow, MFP: milk fat percentage, SCC: somatic cell count, age: month age of cattle at the time of sampling

### Isolation and Identification of Primary BMECs

The composition of the complete culture medium and culture method were the same as those of Duan et al. (2017). When the cells grew to 80% (F_2_ generation), total cellular RNA (Takara, Dalian, China) was extracted. To exclude genomic DNA contamination, DNAase (Takara, Dalian, China) was used for reverse transcriptional preprocessing (Takara, Dalian, China). Primers were designed using Primer Premier 5.0 and PCR amplification was performed for genes specifically expressed in BMECs related to milk fat synthesis and keratin 8 (Takara, Dalian, China).

PCR reaction procedure: pre-denaturation at 94°C for 4 min, denaturation at 94°C for 30 s, annealing (Supplementary Table S2 for annealing temperature) for 30 s, extension at 72°C for 30 s, 35 cycles, 72°C extensions for 10 min.

The basic identification of BMECs is the same as that of Shen et al. (2016). The triglyceride (TAG) content of BMECs was determined by using the triglyceride enzymatic assay kit (Applygen, Beijing, China) of the high-fat samples. Then the protein concentration of the sample was measured by using the BCA protein content detection kit, and the TAG content was adjusted for the protein concentration per mg.

### Library Construction and Transcriptome Sequencing

The Trizol method was used to extract total RNA from BMECs (F2) of high and low MFP cows, and the quality of RNA samples was strictly controlled. The integrity of RNA and DNA contamination were analyzed by agarose gel electrophoresis. The concentration and purity of RNA (OD260/280) were detected by Nanodrop, and the integrity of RNA was accurately detected by Agilent 2100 bioanalyzer. The 260/280 ratio of all samples was approximately 2.0, and the RNA integrity index (RIN) was ≥8.0.

A chain-specific library was constructed by removing ribosomal RNA. First, ribosomal RNA was removed from the total RNA, and RNase R enzyme was used to break RNA into short fragments of 250–300 bp, and cDNA was synthesized. The purified double-stranded cDNA was repaired at the end, added A tail and connected to the sequencing junction. Finally, the library was amplified by PCR and the library was obtained.

After passing the library inspection, Illumina PE150 sequencing was performed. After the original data is obtained, the reads with adapter, N (undetermined base information) ratio greater than 0.002, and low-quality bases with a read length of more than 50% are removed. After sequencing error rates (Q20 and Q30) and GC content distribution checks, clean reads for subsequent analysis were obtained. tophat2 (http://tophat.cbcb.umd.edu), Hisat2 (http://ccb.jhu.edu/software/hisat2), and STAR (http://code.google.com/p/rna-star) software were used to compare and analyze the RNA sequencing (RNA-seq) data (the reference genome version is bos_taurus_Ensembl_97).

### DEGs, DELs Screening, and Functional Enrichment Analysis

The level of gene expression was quantified using fragments per kilobase of exon per millions of reads (FPKM) value. Expression difference significance analysis was performed using edgeR software (Smyth et al., 2010). The corrected *p*-value (Padj) was used to determine the significance level, and Padj <0.05 was used as the standard of differential significance, while |log2FoldChange|>1.5 was another important criterion for screening DELs and DEGs. Co-localization of lncRNAs with protein-coding genes was used to find genes within 100 kb upstream and downstream of lncRNAs, and co-expression was used to predict the target genes of lncRNAs. The gene ontology (GO) database was used to enrich cellular composition (CC), biological process (BP), and molecular function (MF) of DEGs and target genes of DELs. Kyoto encyclopedia of genes and genomes (KEGG) was used to identify the major biochemical metabolic pathways and signal transduction pathways involved in genes. The protein interaction network between DEGs and DELs target genes was analyzed by STRING database (https://string-db.org/) and further visualized using Cytoscape (http://www.cytos-cape.org/).

### MiRNA Target Prediction

MiRanda database was used with the default parameters to identify conserved miRNA target sites in the 3′UTR of lncRNAs (total score ≥140, total energy ≤ -15 kcal/mol; John et al., 2004). MiRNAs target gene prediction was based on the intersection of miRanda (total score ≥140, total energy ≤ -15 kcal/mol) and RNAhybrid (mfe ≤ -20 kcal/mol, *p* < 0.05; John et al., 2004; Jan and Marc, 2006).

### qRT-PCR Validation of Sequencing Data

To confirm the sequencing results, eight DEGs and DELs were randomly selected for qRT-PCR validation, respectively. First-strand cDNA synthesis was performed using the PrimeScript RT kit (Takara, Dalian, China). qRT-PCR (three replicates) was performed by SYBR Premix Ex Taq™II (Takara, Dalian, China) on the Bio-Rad CFX96 Touch™ Real-Time PCR Detection System (Bio-Rad, Hercules, CA, United States). Amplification procedure: 95°C for 30 s, 95°C for 5 s, annealing for 30 s, 40 cycles. qRT-PCR primers were designed using Primer Premier 5.0 with primers spanning at least one intron, and the sequence and annealing temperature of each primer are shown in Supplementary Table S2.

### Data Analysis

The data were filtered by Microsoft Excel 2016, and the relative expressions of DEGs and DELs were analyzed by the 2^−ΔΔCt^ method and normalized by the GAPDH gene. The data were analyzed using SAS 9.2 (SAS Institute, Cary, NC, United States) with a linear mixed model for one-way ANOVA.

## Results

### Isolation, Culture, and Identification of BMECs From Milk

**FIGURE 1:**
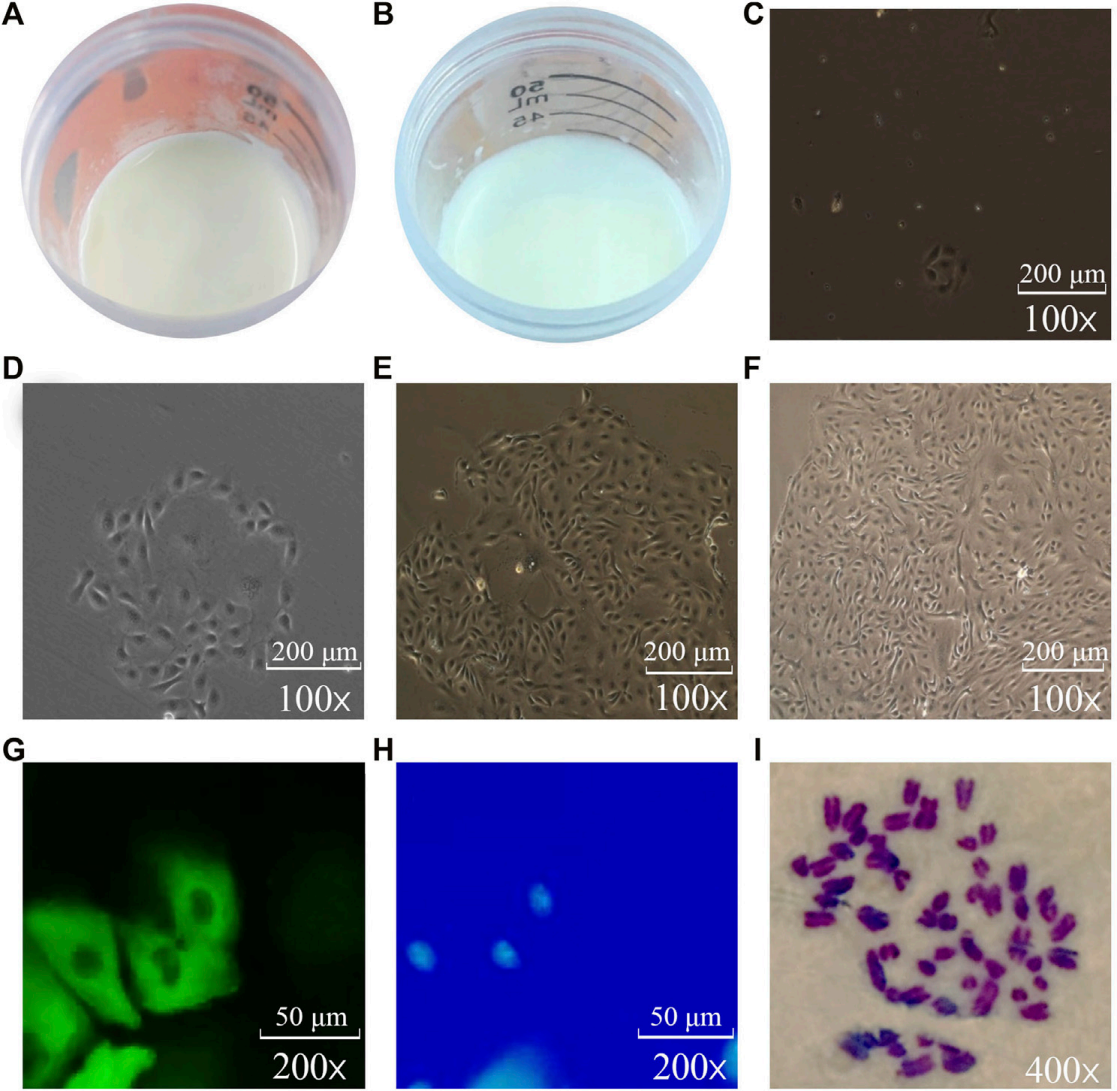
Isolation, culture and identification of BMECs (**C–F**: 10*10; **G, H**: 10*20; **I**: 10*40). **(A)**: Thickness and area of milk fat layer after centrifugation of high-milk fat milk. **(B)**: Thickness and area of milk fat layer after centrifugation of low-milk fat milk. **(C)**: Single-cell clone of BMECs can be seen after 3 days of isolation and culture. **(D)**: Single-cell clones of BMECs gradually expand after 5 days of culture. **(E)**: BMECs have begun to proliferate rapidly after 7 days. **(F)**: After 13 days of isolation and culture, BMECs can be rapidly covered with the bottom of the bottle. **(G)**: BMECs labeled with green fluorescence. **(H)**: Nuclei stained blue by DAPI in the same visual field. **(I)**: Chromosomes under oil microscope.

**FIGURE 2:**
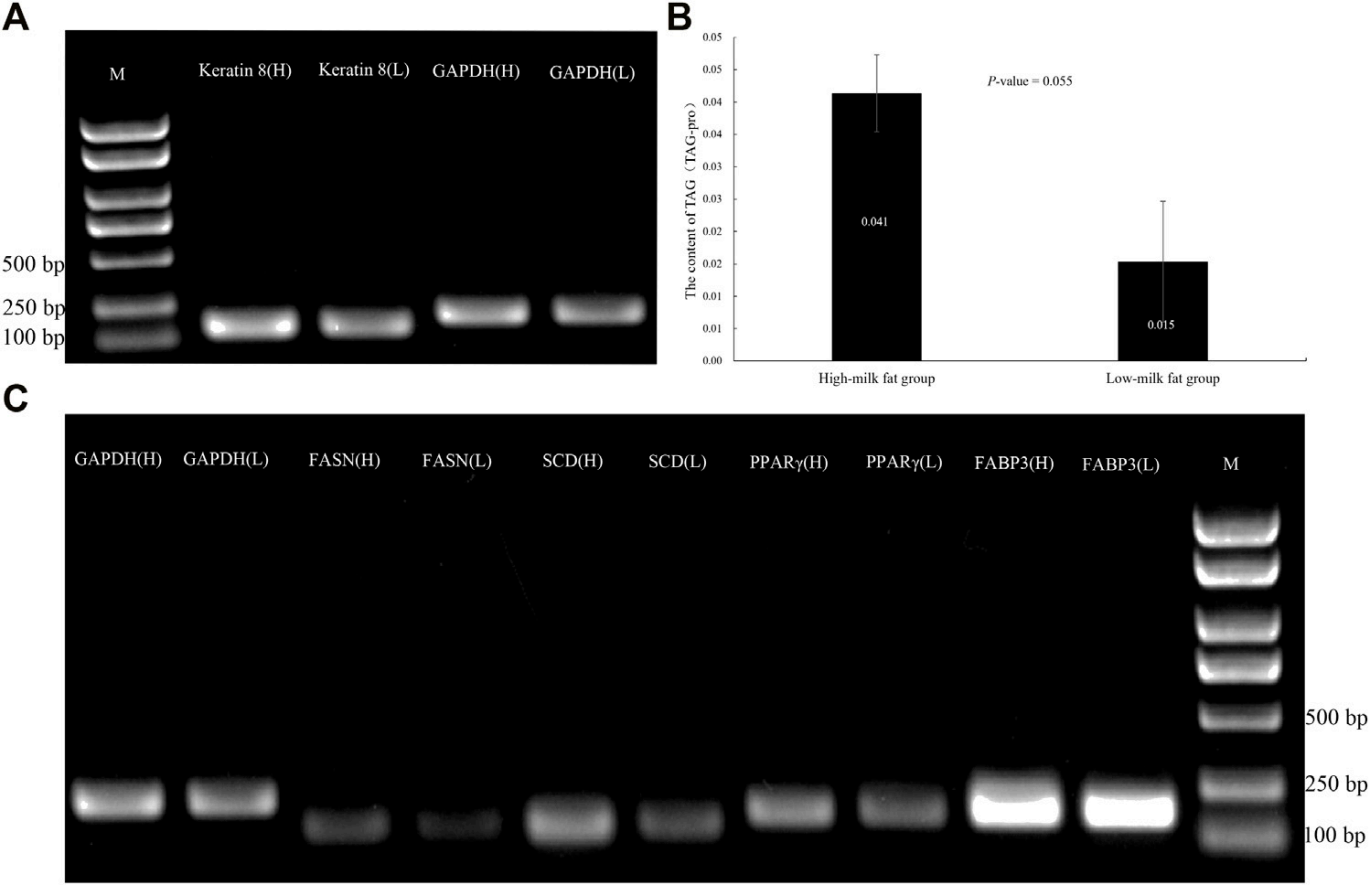
BMECs TAG content and expression level of adipogenic genes and keratin 8 in high and low MFP. **(A)**: Expression of keratin 8 in BMECs of dairy cows with high and low MFP. **(B)**: Content of TAG in BMECs of dairy cows with high and low MFP. **(C)**: Expression of adipogenic genes in BMECs of dairy cows with high and low MFP. M, DL2000 DNA marker, H, high-milk fat group, L, low-milk fat group.

As shown in Figure 1, the fresh milk (per 40 ml) of dairy cows with high MFP and low MFP was centrifuged at 1,000 r/min for 10 min. It is found that the milk fat layer area of high MFP dairy cows are higher than that of low MFP dairy cows, and there are also different milk fat layer thickness (Figures 1A,B). The isolated BMECs is a unique “pebble” epithelial cells shape, and Figures 1C–F is the flow chart of the isolation BMECs, genetics, growth, and cell secretory characteristics are normal (Zheng et al., 2018). The cell growth curve conforms to the “S” rule, indicating that the isolated cells have grown well (Hu et al., 2009). Keratin 18 belongs to the intermediate fibrin family, is participated in the formation of the cytoskeleton, and is the marker of epithelial cells (Hu et al., 2009). In this study, immunofluorescence showed that keratin 18 was positive (Figures 1G,H), and the expression of keratin 8 (epithelial cell-specific protein) was detected in both high and low groups (Figure 2A), indicating that the isolated cells had specific epithelial cell characteristics (Lu et al., 2012). Chromosome analysis showed that the cells were in a normal diploid configuration and contained 60 chromosomes, which was consistent with the chromosome number of dairy cows (Figure 1I).

### BMECs TAG Content and Expression Level of Adipogenic Genes in High and Low Milk Fat Groups

Shen et al. (2016) showed that the isolated BMECs maintained cell-specific genetic, structural, and biological functions in the 2^nd^ and 5^th^ passages. To confirm whether the BMECs isolated from the high-milk fat group and low-milk fat group have the differences in milk fat synthesis at the cellular level, we detected the changes in the TAG content and adipogenic gene expression of F2 generation BMECs. The results found that there was a great difference in TAG content, and the expression levels of fat-related genes *SCD*, *PPAR γ*, and *FABP3* were higher in high-milk fat group than those in low-milk fat group, which further confirmed the difference between high and low MFP groups at the molecular level. It has laid a reliable material foundation for subsequent transcriptome sequencing and other experiments (Figure 2).

### Overview of BMECs Sequencing Data

Using Illumina PE150 sequencing platform, 81 605 996 ∼ 97 102 888 and 78 710 246 ∼ 88 676 080 raw_reads were obtained in high and low MFP BMECs, and 80 633 532 ∼ 94 731 948 and 76 807 276 ∼ 86 508 476 clean_reads were obtained after removing the adapter related, containing N and low quality, respectively. The sequencing error rate of the 8 samples is 0.02%, Q20 is greater than 97%, Q30 is greater than 94%, the GC content is about 53%, which ensures the accuracy of the subsequent analysis. The genomic distribution of clean_reads showed that most of the clean_reads were located in the exon region (61.79–70.27%), the intergenic region (20.96–24.41%), and the intron region (8.03–14.46%) of the bovine genome.

**FIGURE 3:**
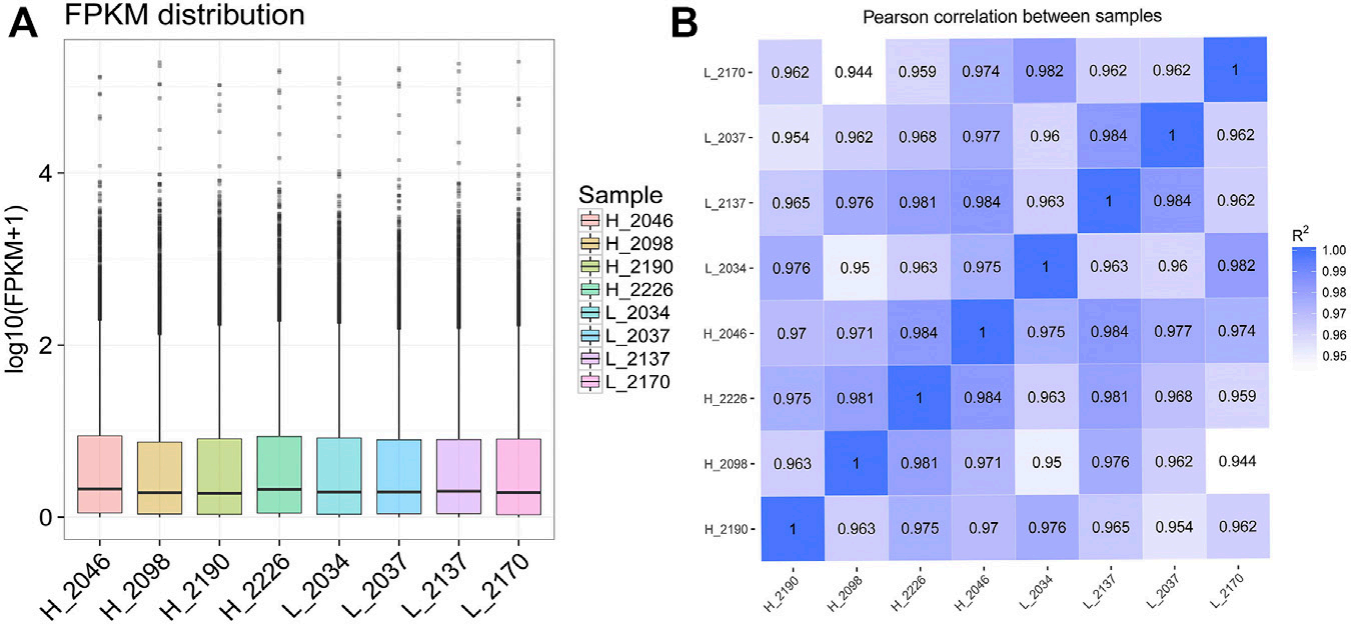
Expression level distribution and correlation analysis of each sample. **(A)**: The box diagram of different sample expression values, in which the abscissa is the sample name and the ordinate is log10 (FPKM + 1). The box diagram of each region corresponds to five statistics (from top to bottom, the maximum, upper quartile, median, lower quartile, and minimum). **(B)**: The heat maps for the correlation analysis between samples. FPKM, Fragments per kilobase of exon per million fragments mapped, R^2^, Pearson correlation coefficient.

CPC/PFAM/CNCI software predicted that there were 25,702 novel_mRNA with coding potential and 7602 candidate novel_lncRNAs without coding potential. Among all the candidate novel_lncRNAs, the lncRNAs located in the intergenic region account for 51.8%, the antisense contains 24.75%, the sense overlapping contains 23.50%, and no sense intronic. After obtaining the FPKM of all transcripts, the distribution of transcripts expression levels of different samples was shown by block diagram (Figure 3A), indicating that the BMECs expression level in cows with high MFP was slightly higher than that in cows with low MFP. The correlation of gene expression level between samples is an important index for testing the experimental reliability and sample selection. In this study, the correlation coefficients of the intra-group and inter-group samples were calculated based on the FPKM values of all genes in each sample and drawn into a heat map (Figure 3B). As can be seen from the figure, intra-group correlation coefficients are all above 0.96, graeter than the ideal sampling and experimental conditions (*R* ^2^ = 0.92), indicating a high degree of similarity in expression patterns among samples and a difference between groups. Therefore, we think that the transcriptome sequencing results are reliable and can be used for subsequent analysis.

### Differential Expression and Clustering Analysis of DEGs and DELs

**FIGURE 4:**
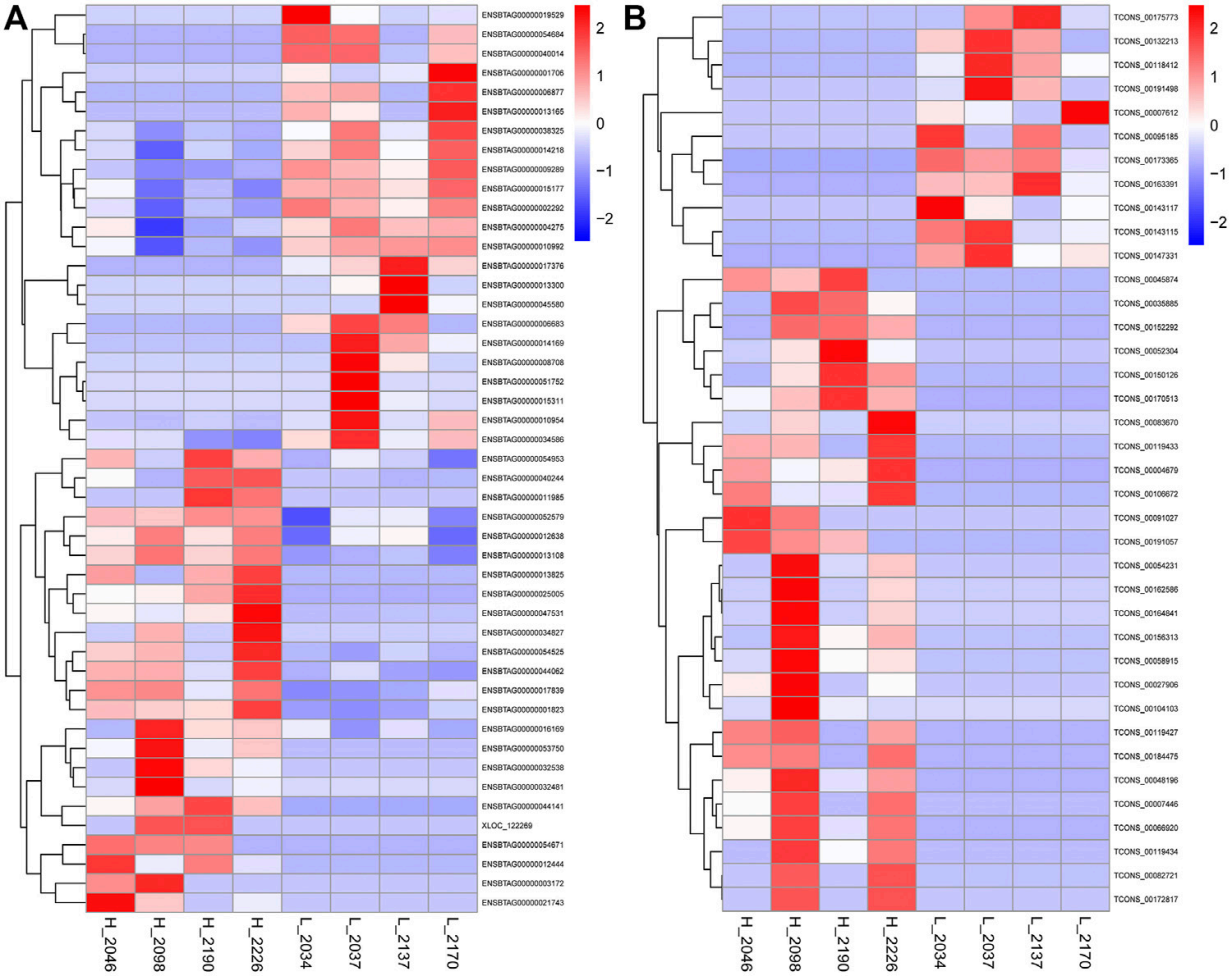
Hierarchical clustering of DEGs and DELs. **(A)**: The DEGs clustering heat map, the abscissa is the sample, the ordinate is the DEGs, the left side clusters the genes according to the degree of similarity of expression, the expression is gradually upregulated from blue to red, and the number is the relative expression after homogenization. **(B)**: The DELs clustering heat map.

Fifteen thousand five hundred and seventy-three mRNAs were common to the high and low milk fat groups, with 717 and 614 mRNAs specifically expressed in the high and low milk fat groups for the differential mRNAs screened, respectively. However, the number of lncRNAs shared between the high-milk fat group and the low-milk fat group was relatively small (4,666), with 372 specific lncRNAs in the high-milk fat group and 365 lncRNAs in the low-milk fat group. A total of 47 DEGs and 38 DELs were screened from BMECs with high and low MFP (Padj <0.05). The FPKM of DEGs and DELs were transformed by log_10_ (FPKM+1) and then clustered by heat map (Figure 4). DEGs or DELs with similar expression patterns cluster together and differ significantly in color, indicating that these genes share a common function or are involved in a common metabolic pathways. Compared with the low-milk fat group, 24/27 DEGs/DELs expression in the high-milk fat group was significantly upregulated, and 23/11 DEGs/DELs expression was significantly downregulated.

### DEGs Enrichment Analysis and Screening of DEGs Related to Lipid Metabolism

**FIGURE 5:**
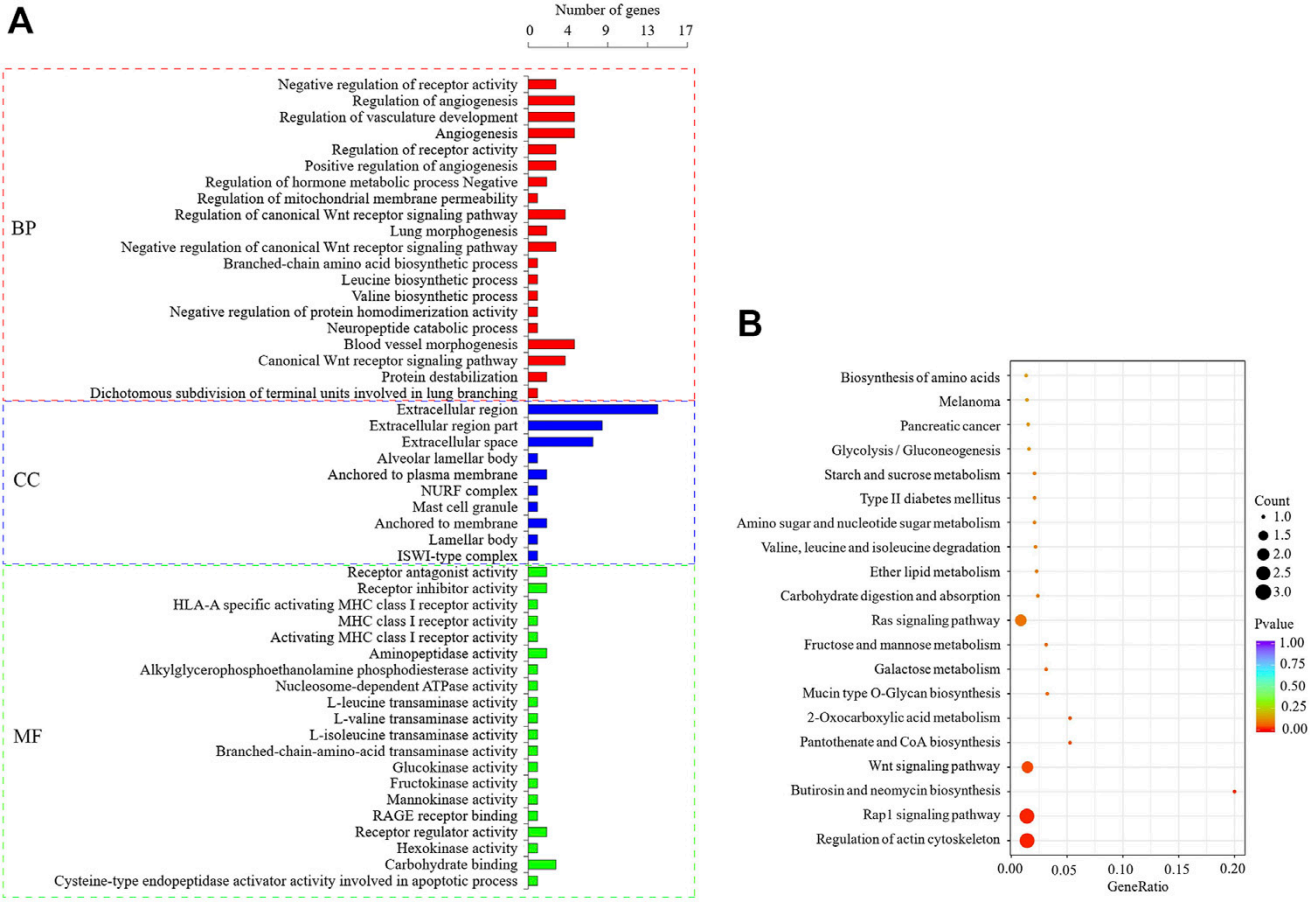
GO and KEGG enrichment analysis of DEGs. **(A)**: The first 20 significant GO items (*p* < 0.05), when less than 20, only show significant enrichment items, the latter is the same. **(B)**: The first 20 KEGG enrichment results.

Based on the functional enrichment analysis of 47 DEGs, a total of 173 significantly enriched GO items were identified, as shown in Figure 5A, showing the first 20 significantly enriched GO items (the same below). Among the significantly enriched GO items, the BP related to lipid metabolism is the regulation of phospholipid translocation, positive regulation of phospholipid translocation, and glycerophospholipid catabolic process. The MF item is triglyceride lipase activity, lipase activity, aminophospholipid transporter activity, phosphodiesterase I activity, sterol esterase activity, and carboxylic ester hydrolase activity. KEGG enrichment analysis showed that six signaling pathways were significantly enriched (Figure 5B), and the pathways related to lipid metabolism were Wnt, Rap1 and 2-Oxocarboxylic acid metabolism. 11 DEGs that may regulate milk fat metabolism were screened out (Table 3). The enrichment of GO/KEGG is shown in Supplementary Table S3. *PDGFD*, *BCAT1*, and *APOL3* genes expression were upregulated, while *ATP8A2*, *PTPRR*, *KCNMA1*, *ZFYVE28*, *ENPP2*, *DKK1*, *CES4A*, and *CTSH* genes expression were downregulated. *ENPP2* gene is significantly enriched to BP, MF, and KEGG pathway related to lipid metabolism, and it is inferred that it is more likely to regulate milk fat metabolism.

**TABLE 3:** Candidate DEGs related to milk fat metabolism.

Gene name	Gene location	Log2FoldChange	Up/down	Padj
PDGFD	15:4259082-4536080	12.0848	Up	0.0364
BCAT1	5:85057073-85174596	11.5674	Up	0.0038
APOL3	5:74600517-74612469	4.8252	Up	0.0089
ATP8A2	12:33380874-33831448	−13.0109	Down	0.0008
PTPRR	5:42611538-42884729	−12.1701	Down	0.0434
KCNMA1	28:32616314-33387186	−12.1505	Down	0.0390
ZFYVE28	6:116265797-116364222	−10.5076	Down	0.0251
ENPP2	14:80982673-81117148	−10.2255	Down	0.0403
DKK1	26:6846290-6849518	−4.3134	Down	0.0061
CES4A	18:34675658-34690406	−2.8026	Down	0.0133
CTSH	21:25201460-25222224	−1.6301	Down	0.0119

Note: Log2FoldChange is logarithm of fold change with a base of 2, Padj is the corrected significance test probability value, gene location is the specific location of the gene on the chromosome.

### Target Genes Prediction and Functional Analysis of DELs

**FIGURE 6:**
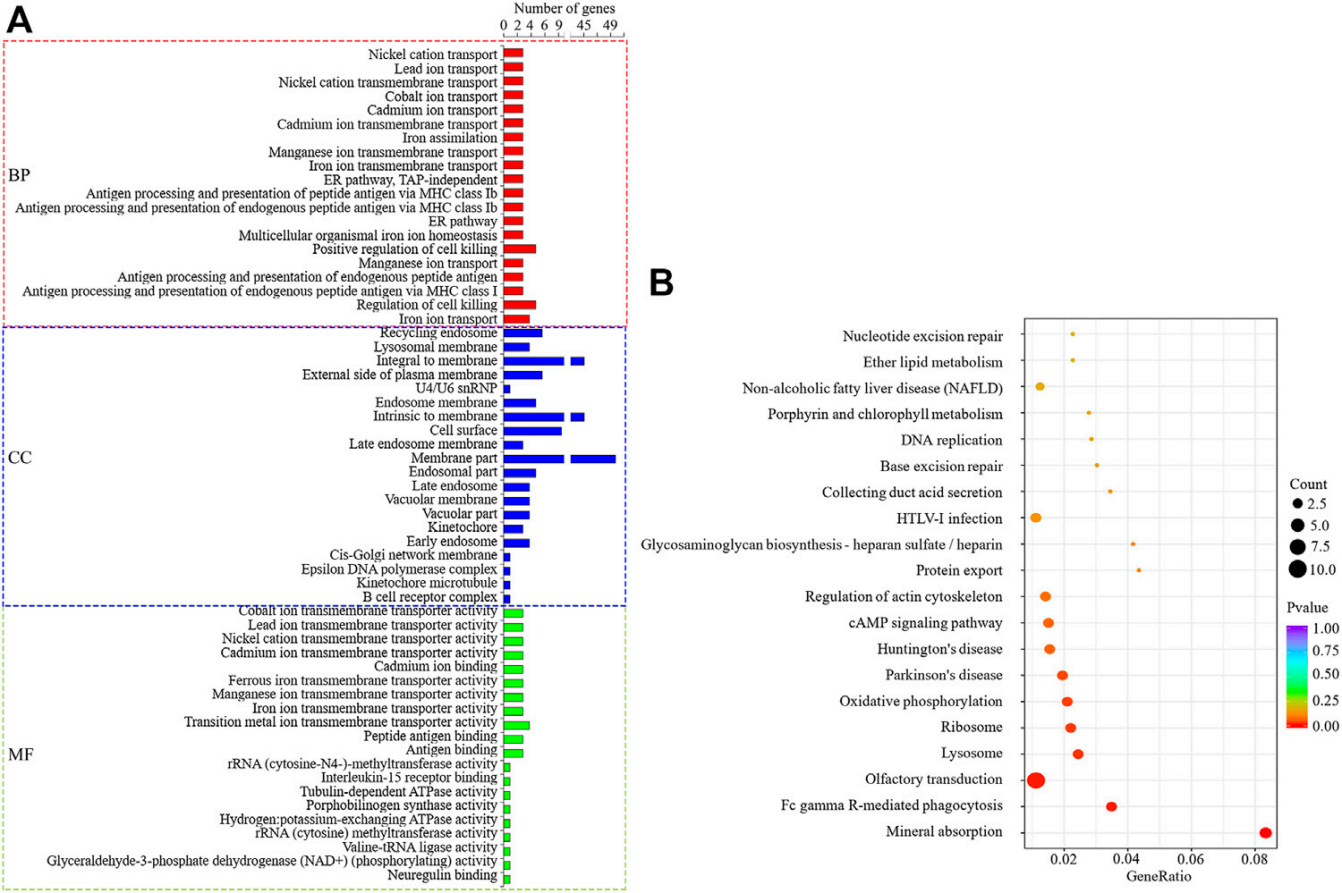
GO and KEGG enrichment analysis of DELs target genes predicted by co-localization. **(A)**: The first 20 significant GO items (*p* < 0.05). **(B)**: The first 20 KEGG enrichment results.

The target genes (cis) of DELs predicted by co-localization were enriched to 258 significant GO items (*p* < 0.05), as shown in Figure 6A. In the significantly enriched GO items, the BP is related to lipid metabolism that regulates the secretion of arachidonic acid, and MF is lipid phosphatase activity. KEGG enrichment analysis showed that seven signal pathways were significantly enriched (Figure 6B), among which Fc gamma R-mediated phagocytosis was related to lipid metabolism. Through enrichment analysis, seven candidate target genes that may regulate milk fat metabolism were screened, namely *VARS2*, *ITGA6*, *ATP4A*, *PPAP2C*, *FGF1*, *AMPH*, and *SYK*. The enrichment of GO/KEGG is shown in Supplementary Table S4.

**FIGURE 7:**
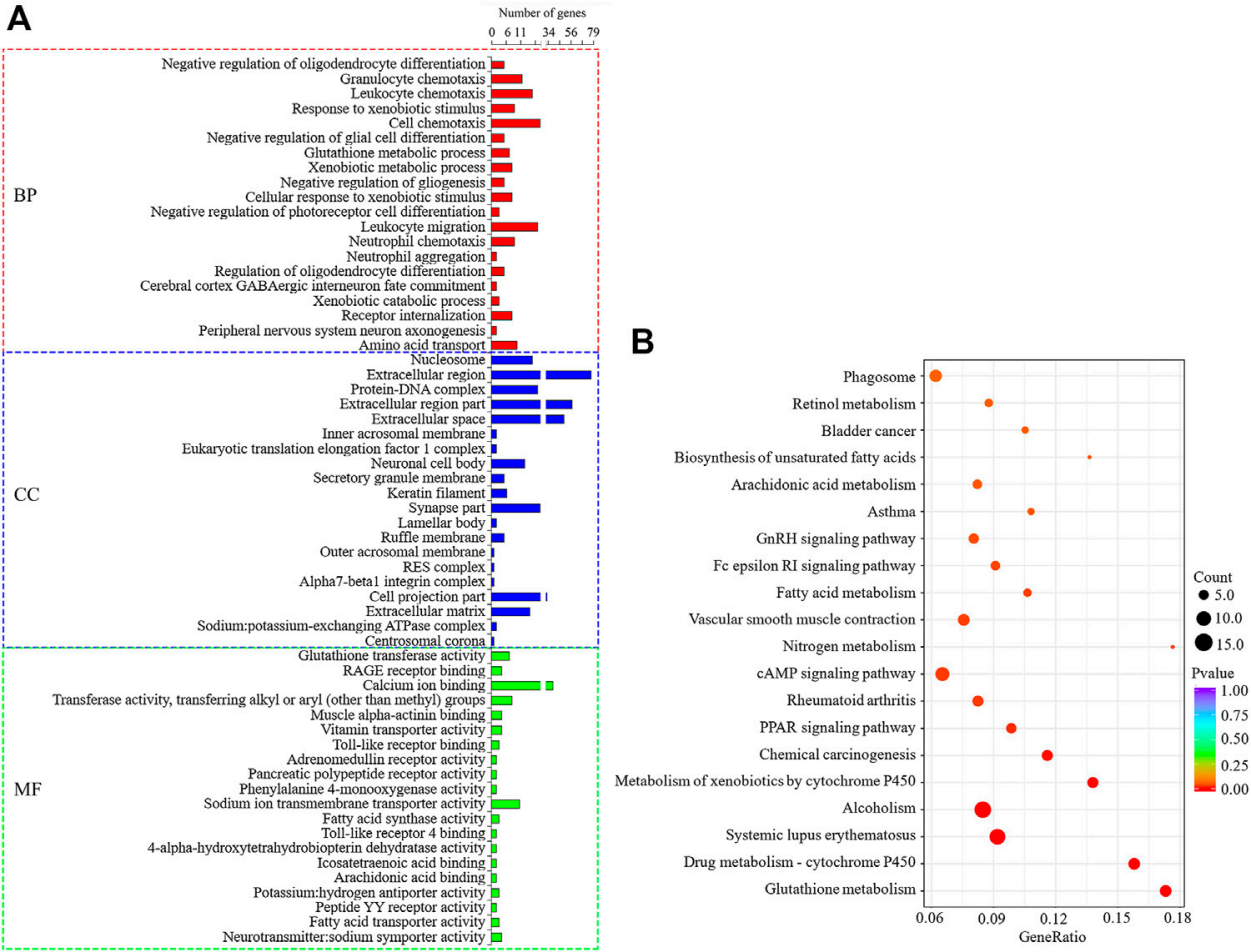
GO and KEGG enrichment analysis of DELs target genes predicted by co-expression. **(A)**: The first 20 significant GO items (*p* < 0.05). **(B)**: The first 20 KEGG enrichment results.

The co-expression predicted DELs target genes (trans) were significantly enriched to 509 GO items (*p* < 0.05), as shown in Figure 7A. Among them, BP related to lipid metabolism includes lipid metabolic process, long-chain fatty-acyl-CoA biosynthetic process, fatty acid extension, fatty acid transport and negative regulation of triglyceride biosynthetic process, and so on. MF items includes fatty acid synthase activity, fatty acid transporter activity, long-chain fatty acid-binding, and lysophospholipid transporter activity, and so on. The target genes were significantly enriched to 17 signal pathways (*p* < 0.05) (Figure 7B), among which the pathways related to lipid metabolism include PPAR, cAMP, Fatty acid metabolism and Fc epsilon RI. Under the condition that the correlation coefficient between DELs and target gene expression was greater than 0.97, 10 target genes that might regulate milk fat metabolism were screened out, namely *PLA2G4E*, *INPP4B*, *FABP3*, *ACADSB*, *FABP4*, *OXSM*, *FABP7*, *GPX1*, *CYP27A1*, and *ALOX12* genes. The GO/KEGG enrichment is shown in Supplementary Table S5. Among the 10 lipid metabolism-related target genes, the gene expression of *PLA2G4E*, *FABP4*, *FABP3*, and *ACADSB* was upregulated (*p* < 0.05), while the gene expression of *FABP7* and *CYP27A1* was downregulated (*p* < 0.05). The upregulation genes of *PLA2G4E*, *FABP4* and *OXSM* is significantly enriched in lipid metabolism-related BP, MF and KEGG. Therefore, these three genes and their corresponding DELs need more attention in the follow-up functional mechanism research.

The 17 lipid metabolism-related target genes were predicted and screened by co-localization and co-expression. All DELs that regulate these target genes are shown in Table 4. A total of 18 DELs that may be related to milk fat metabolism were screened and their corresponding relationships with target genes are shown in Supplementary Tables S6, S7. Among the 18 DELs that may be related to milk fat metabolism, 12 were upregulated and six were downregulated. Bold font is the DELs predicted by both co-localization and co-expression that may regulate milk fat metabolism. Italic is the DELs corresponding to the target genes (*PLA2G4E*, *FABP4*, and *OXSM*), which is significantly enriched in BP, MF, and KEGG. These are also the lncRNAs that need more attention in further experiments.

**TABLE 4:** DELs related to lipid metabolism predicted by co-expression and co-localization.

Transcript id	LncRNA type	Transcript location	Log2FoldChange	Up/down	Padj
**TCONS_00104103**	**sense_overlapping**	**23:27943191-28058062**	**14.3242**	**Up**	**0.0452**
**TCONS_00162586**	**sense_overlapping**	**7:42726724-42986751**	**13.9441**	**Up**	**0.0489**
***TCONS_00172817***	***antisense***	***8:86876741-87001036***	***13.9264***	*Up*	***0.0460***
***TCONS_00082721***	***lincRNA***	***2:24262990-24300737***	***12.7310***	*Up*	***0.0132***
**TCONS_00143117**	**antisense**	**4:82502392-82533931**	**−12.4167**	**Down**	**0.0321**
TCONS_00150126	sense_overlapping	5:28723371-28948902	15.3298	Up	0.0005
*TCONS_00156313*	*antisense*	*6:106460016-106540196*	*14.2909*	*Up*	*0.0039*
TCONS_00164841	antisense	7:102898847-103228659	14.0298	Up	0.0455
TCONS_00119434	lincRNA	27:36076831-36107350	12.7668	Up	0.0206
TCONS_00027906	antisense	12:28946242-29075799	12.6328	Up	0.0270
TCONS_00054231	antisense	16:45117041-45146121	12.2759	Up	0.0244
TCONS_00066920	sense_overlapping	18:46165575-46169765	11.5972	Up	0.0086
*TCONS_00058915*	*sense_overlapping*	*17:6204238-6224363*	*10.2082*	*Up*	*0.0437*
TCONS_00191498	lincRNA	X:124180006-124280661	−13.9321	Down	0.0077
TCONS_00143115	antisense	4:82475604-82533958	−12.4900	Down	0.0025
*TCONS_00007612*	*lincRNA*	*1:95512675-95550502*	−*12.1321*	*Down*	*0.0421*
TCONS_00118412	lincRNA	27:16563-41295	−11.9714	Down	0.0043
TCONS_00163391	lincRNA	7:53750360-53759144	−10.6626	Down	0.0182

Note: Italic is the DELs, corresponding to the target genes (*PLA2G4E*, *FABP4*, and *OXSM*), which is significantly enriched in BP, MF, and KEGG., Bold font is the DELs, predicted by both co-localization and co-expression that may regulate milk fat metabolism. transcript location is the specific location of the DELs, on the chromosome. Padj is the corrected significance test probability value. Bold-italic are DELs corresponding to target genes (*PLA2G4E*, *FABP4*, and *OXSM*) that were significantly enriched in BP, MF and KEGG. Bold are differential genes related to milk fat metabolism predicted by co-localization and co-expression.

**FIGURE 8:**
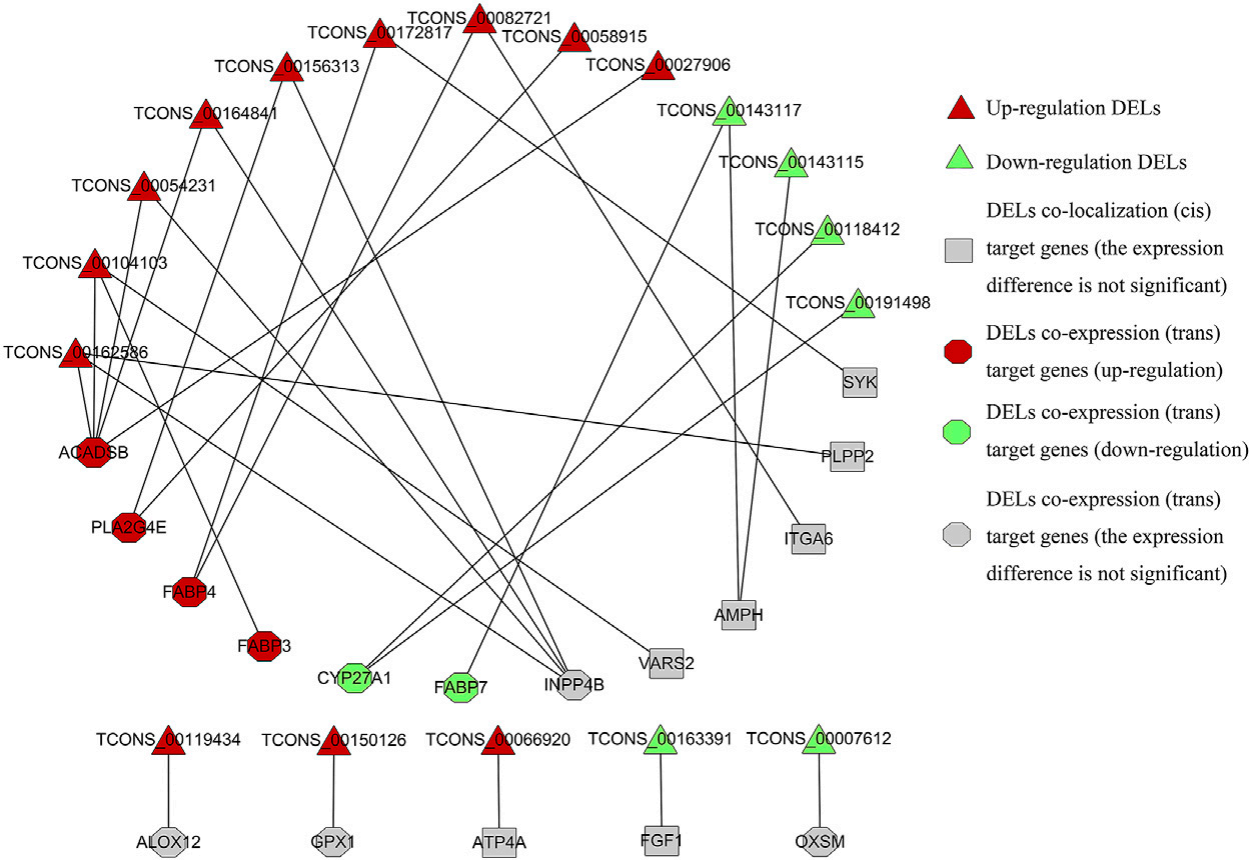
Regulatory relationship between DELs and target genes related to lipid metabolism.

The interaction between 18 DELs and lipid metabolism-related target genes is shown in Figure 8. A lncRNA may interact with multiple lipid metabolism-related genes, and a gene may also be regulated by multiple lncRNAs. Among the target genes related to lipid metabolism, the expression trend of significantly upregulated or downregulated genes is consistent with the targeted regulation of DELs, which is not only in line with the general mode of action of lncRNAs on genes but also the focus of subsequent experiments.

### Construction of lncRNA_miRNA_mRNA Interaction Regulation Network

**FIGURE 9:**
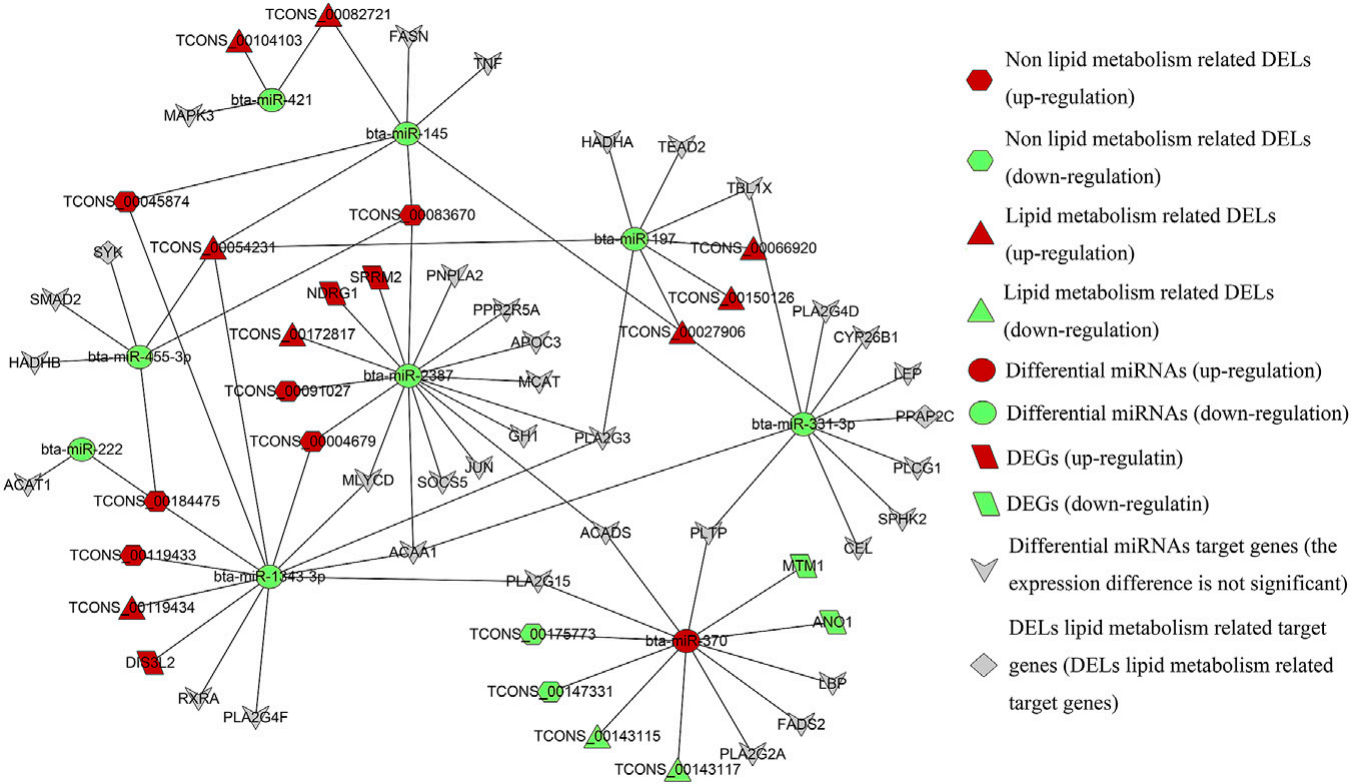
Regulatory network of DELs differential miRNAs_ target genes.

The differential miRNAs used in this section were derived from the small RNA sequencing results of the research team (Supplementary Table S8). LncRNAs can act as precursor molecules of miRNAs. When looking for lncRNAs that interact with miRNAs, it is necessary to filter out lncRNAs that may be precursors of miRNAs. Based on the homology between lncRNAs and miRNAs precursors, we use Blastn software to search for such lncRNAs, and the results are listed in Supplementary Table S9. Then, the lncRNAs corresponding to differential miRNAs were predicted by miRanda software, and miRNAs regulated by the DELs are screened. The target genes of differential miRNAs were predicted and cross-differentiated by using two software, miRanda, and RNAhybrid. Based on ceRNA theory, we searched the lncRNAs_target gene pairs with the same miRNAs binding sites, construction of lncRNA_miRNA _mRNA regulatory relationship with lncRNAs as a decoy, miRNAs as core and mRNAs as the target, and calculation of Pearson correlation coefficients for the expression of lncRNA_miRNA and miRNA_mRNA (Figure 9).

As shown in the regulatory network diagram, all lipid metabolism-related target genes *SYK* and *PPAP2C* (cis) are also the target genes of lipid metabolism DELs, constituting the regulatory relationships of TCONS_00054231_bta-miR-455-3p_*SYK*, TCONS_00184475 _bta-miR-455-3p_*SYK*, and TCONS_00027906_bta-miR-331-3p_*PPAP2C*. The upregulated genes *DIS3L2*, *SRRM2*, *NDRG1* and the downregulated genes *ANO1* and *MTM1* are the DEGs screened in this study, and 18 ceRNAs regulatory networks contain these DEGs. Also, lipid metabolism-related DELs are involved in a total of 76 ceRNAs regulatory networks, including 10 lncRNAs, 8 miRNAs, and 32 genes. There are 80 ceRNAs regulatory networks involved in lipid metabolism-unrelated DELs, consisting of 8 lncRNAs, six miRNAs, and 24 genes, are enriched for target genes GO/KEGG as shown in Supplementary Table S10. Downregulated bta-miR-2387 is a differential miRNA with the largest number of potential lipid metabolism-related target genes (13) and only interacts with lipid metabolism-related differential lncRNA TCONS_00172817. Non-lipid metabolism-related differences lncRNA TCONS_00004679, TCONS_00091027, and TCONS_00083670 may all play a regulatory role in bta-miR-2387.

It has been shown that the downregulation of bta-miR-145 among many differential miRNAs can regulate the lipid droplet formation and TAG accumulation (Wang et al., 2017). Therefore, the regulatory relationship centered on bta-miR-145 is one of the focuses of our subsequent experiments. Notably, TCONS_00027906, TCONS_00054231, TCONS_ 00082721, TCONS_00045874, and TCONS_00083670 act together on bta-miR-145, which in turn regulates the expression of *FASN*, which was a potential regulatory network for probing the mechanism of milk fat regulation of the potential regulatory network. The downregulated expression of bta-miR-421, bta-miR-331-3p and bta-miR-197 was a lipid metabolism DELs that specifically targets lipid metabolism-related differential miRNAs, while bta-miR-222 was a non-lipid metabolism differential lncRNA specifically targets lipid metabolism-related differential miRNAs. Interestingly, among the numerous lipid metabolism-related differential miRNAs, only bta-miR-370 is an upregulated differential miRNA, while the rest are downregulated.

Within the entire regulatory network relationship, the differential miRNAs were negatively correlated with DELs expression in all samples (8) with a correlation coefficient of -0.10 to -0.75. There was a negative correlation between differential miRNAs and the expression of some target genes (-0.10 to -0.67). e.g.: bta-miR-370 with *MTM1*, *ANO1*, *PLA2G15*, *FADS2* genes; bta-miR-2387 with *SRRM2*, *NDRG1*, *PPP2R5A* genes; bta-miR-455-3p with *SMAD2*, *HADHB* genes; bta-miR-1343-3p with *RXRA*, *DIS3L2*, *PLA2G4F* genes; bta-miR-331-3p with *PLTP*, *SPHK2*, *LEP*, *CYP26B1*, *TBL1X* genes; bta-miR-197 with *TBL1X* genes (Supplementary Table S11). We need to focus on the lncRNA_miRNA_mRNA interaction regulatory network with negative correlation and targeted relationship in the subsequent experiments.

### Protein Interaction Network Analysis

**FIGURE 10:**
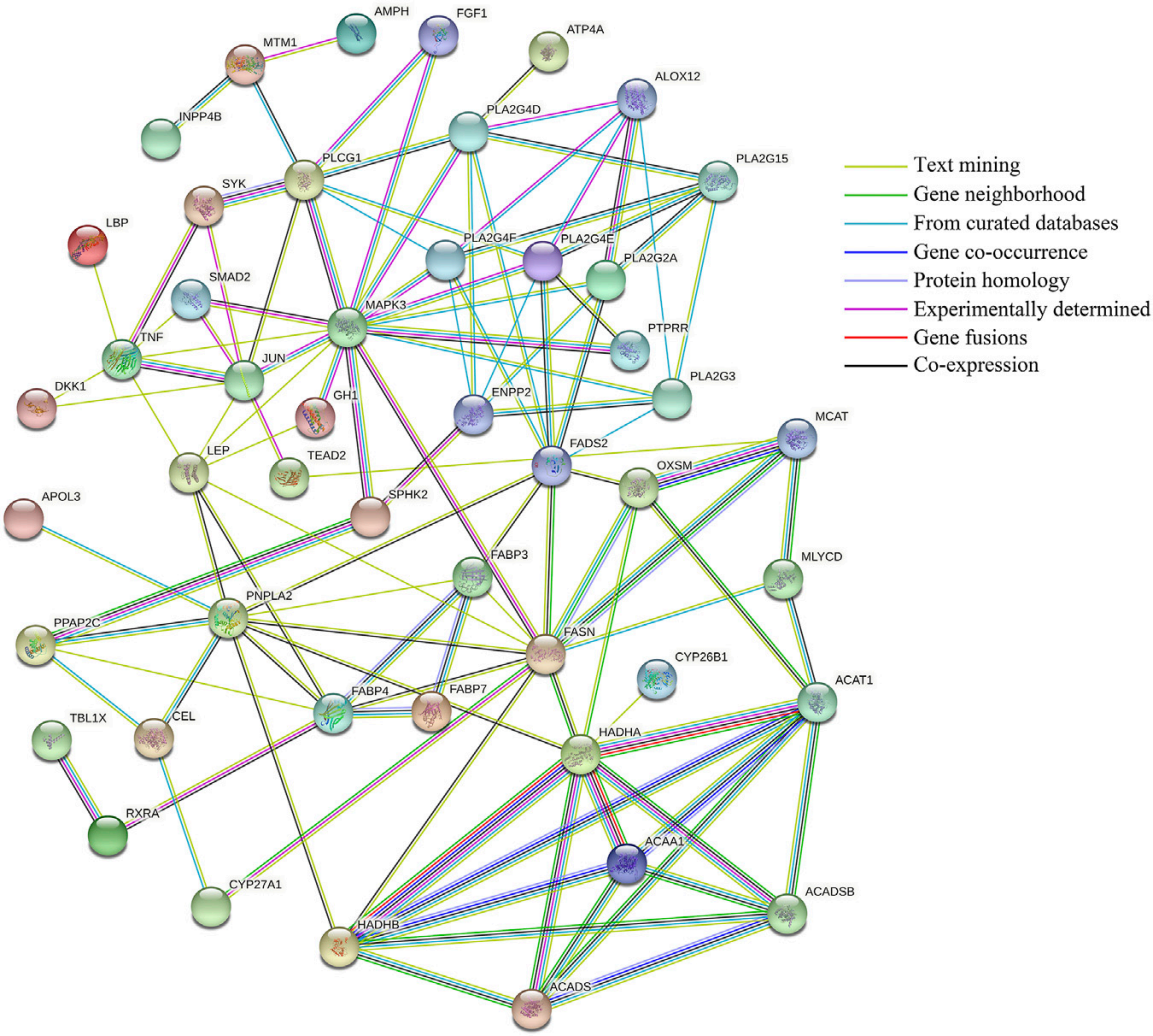
Protein interaction network analysis. Lines of different colors indicate different supporting evidence for protein interactions.

Protein interaction network analysis of DEGs (11) screened the possible regulation of milk lipid metabolism, target genes predicted by the co-localization and co-expression of DELs (17), and lipid metabolism-related genes corresponding to differential miRNAs targeted by DELs (34), the results are shown in Figure 10. There are 49 nodes and 113 interaction link sets in the protein interaction network diagram, with an enriched *p*-value of 1.0e-16 and a confidence level of 0.4 (moderate). The different colored bonds represent the source of evidence for protein interactions, and the more bonds involved in biological functions, the more likely they are. The *PLTP*、*PPP2R5A*、*SOCS5*、*APOC3*、*VARS2*、*ITGA6*、*PDGFD*、*BCAT1*、*ATP8A2*、*KCNMA1*、*ZFYVE28*、*CES4A* and *CTSH* genes are not represented in the figure because they do not interact with each other or with other genes. The interaction among *HADHA*、*ACAT1*、*ACAA1*、*HADHB*、*ACADS* and *ACADSB*, *FABP3*, *FABP4*, and *FABP7*, *FASN*, *OXSM*, and *MCAT* are strongly supported by the data with very high intensity as can be seen from the figure. Secondly, *SYK* and *PLCG1*, *TNF* and *JUN*, *PPAP2C* and *SPHK2*, *TBL1X* and *RXRA* may also have shared functions between genes. Notably, *MAPK3* has a strong interaction with several lipid metabolism-related genes (*JUN*, *SMAD2*, *GH1*, *SPHK2*, *FASN*, *PTPRR*, *PLA2G4E*, *PLA2G4F*, *PLA2G4D*, *FGF1*, and *PLCG1*), respectively, and may jointly play an important role in regulating milk lipid metabolism. The remaining genes also interacted with each other. Although the data support is relatively small, they still have some reference value.

### The Expression Levels of DELs and DEGs Were Verified by qRT-PCR

**FIGURE 11:**
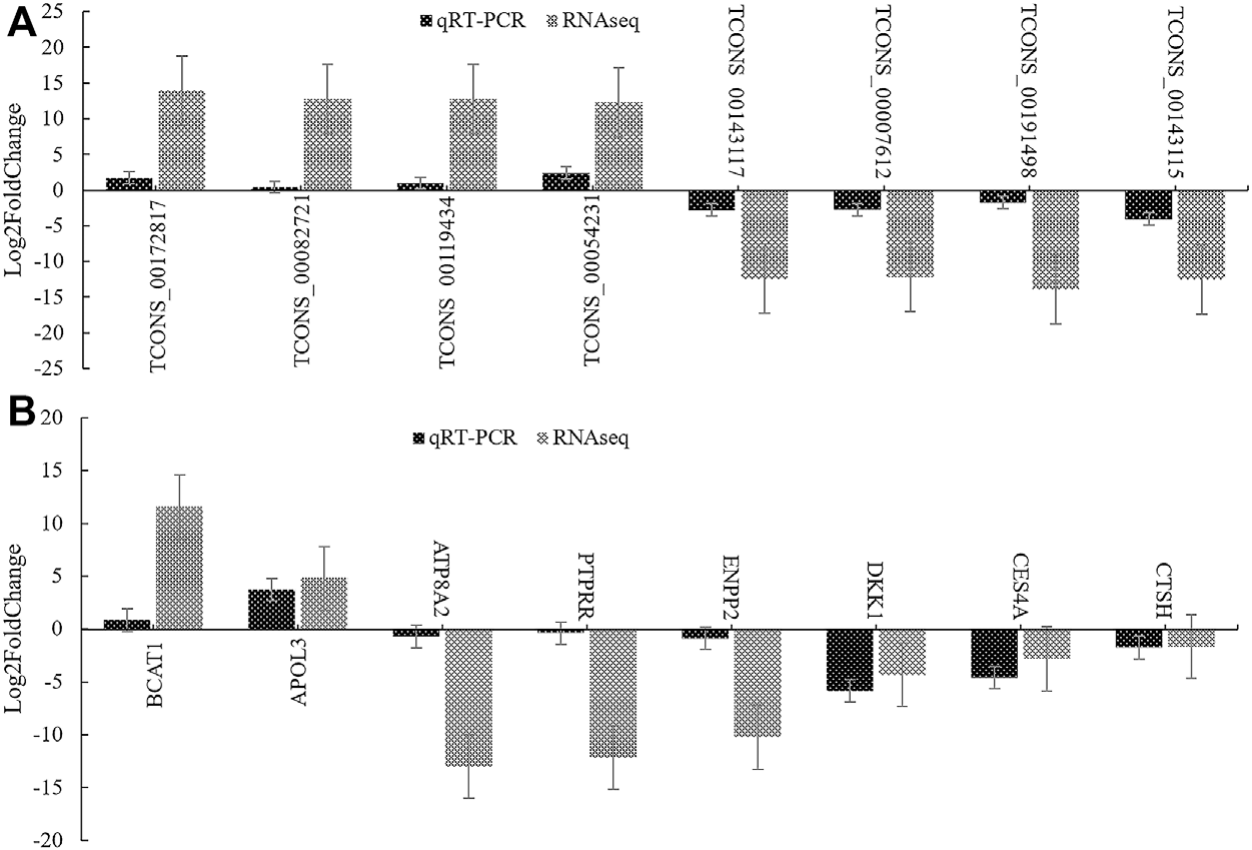
qRT-PCR verification of DEGs and DELs. **(A)**: The verification result of DELs. **(B)**: The verification result of DEGs. log2FoldChange is logarithm of fold change with a base of 2.

We randomly selected 8 DELs and 8 DEGs, analyzed their relative expression levels by qRT-PCR in high and low groups (three repeats), and converted the difference multiplea by log_2_FoldChange. As shown in Figure 11, the results of the qRT-PCR were consistent with those of RNA-seq, which confirmed the accuracy of transcriptome sequencing.

## Discussion

### Candidate DEGs and DELs in Milk Fat Metabolism

It has been confirmed that many genes are involved in the lactation initiation, maintenance and growth and development of the mammary gland through direct or indirect regulation, such as ErbB3, IL-8 and FGF2 (Bach et al., 2017; Williams et al., 2017; Dai et al., 2018). In this study, a total of 11 candidate DEGs related to lipid metabolism were screened and enriched in PI3K-Akt, MAPK, and Wnt lipid metabolism-related pathways (Kessenbrock et al., 2017; Bing et al., 2018). The downregulated *ENPP2* gene is significantly enriched in BP, MF, and lipid metabolism-related pathways, and it has been shown that the lack of *ENPP2* has a significant protective effect on hepatic steatosis, suggesting a possible role of ENPP2 gene in the metabolism of milk lipids (Brandon et al., 2019). Dong et al. (2020) found that the expression of *PDGFD* in adipose tissue of obese individuals was higher than that of lean individuals. In this study, *PDGFD* was also highly expressed in BMECs with high MFP; meanwhile, the downregulated gene *KCNMA1* also showed a downregulated trend in white adipose tissue and hypertrophied adipocytes of mice on the high-fat diet (Nishizuka et al., 2016), which confirmed the reliability of sequencing, suggesting that *PDGFD and KCNMA1* may play an important role in milk fat metabolism. *BCAT1* is a branched-chain aminotransferase that has been shown to mainly affect MFP, milk protein percentage, and milk yield of dairy cows (Fang et al., 2014). *CES4A* has also been shown to be mainly involved in lipid metabolism (Holmes et al., 2010). However, how *BCAT1* and *CES4A* regulate milk fat synthesis has not yet been reported This will be the focus of our future research. It is worth noting that the latest literature reportes that the differentially downregulated *DKK1* related to lipid metabolism seems to play a significant role in promoting obesity in mice (Colditz et al., 2020), which is inconsistent with the results of this study. It may be related to different species or tissues in the study. Other lipid metabolism-related DEGs *APOL3*, *ATP8A2*, *PTPRR*, *ZFYVE28*, and *CTSH* have been reported to associate with kidney disease (Pays, 2021), nerve disease (Choi et al., 2019), ovarian cancer (Wang et al., 2019), glomerular filtration barrier (Zambrano et al., 2018) and atherosclerosis (Lim and Chow, 2006).

LncRNAs can regulate gene expression within 100 kb (cis), and can also change the expression of distant mRNAs (trans) (Guttman et al., 2009). The functional enrichment analysis of 38 target genes of DELs showed that 17 target genes (10 co-expression and seven co-localization) were enriched in lipid metabolism-related pathways. Among them, *FABP3*, *FABP4*, and *FABP7* are *trans*-target genes. Studies have confirmed that these genes encode highly abundant fatty acid-binding proteins in bovine mammary glands and transport long-chain fatty acids to the endoplasmic reticulum to synthesize TAG (Bionaz and Loor, 2008). Protein interaction network analysis showed that the interactions between *FABP3*, *FABP4*, and *FABP7* were also strongly supported by data, which further indicated that they may have similar functions and participate in common BP.

Based on bioinformatics analysis, a total of 18 candidate DELs that may affect milk fat synthesis in dairy cows were screened out. A lncRNA may target multiple genes related to lipid metabolism, and a gene may be regulated by multiple lncRNAs (Zheng et al., 2018). These results indicated that lncRNAs were diverse in regulating the function of target genes. In addition, some of the lipid metabolism-related target genes are also significantly upregulated or downregulated. The target genes expression trends are consistent with the DELs that may be targeted and regulated, which is consistent with the general mode of action of lncRNAs on genes (Cesana et al., 2011), and further reflects the accuracy of the sequencing results. It is worth noting that *FABP4* is a gene encoding a highly rich fatty acid-binding proteins in the mammary gland, which may have a targeting relationship with TCONS _00082721 and TCONS _00172817 (Dong et al., 2020). Among all lncRNAs related to lipid metabolism, TCONS_00082721, TCONS_00119434, TCONS_00191498, TCONS_00007612, TCONS_ 00118412, and TCONS_00163391 are long intervening/intergenic noncoding RNAs (lincRNAs). Because lincRNAs do not overlap with exon sequences in organisms and show a higher degree of tissue specificity (Moran. et al., 2011) and disease specificity (Iyer et al., 2015). They are involved in chromatin modification, epigenetic regulation, genomic imprinting, transcriptional control, and mRNA processing before and after translation (Ulitsky and Bartel, 2013). Therefore, lincRNAs related to milk fat metabolism will be the focus of our subsequent studies. The lncRNAs that might regulate milk fat metabolism are predicted by co-localization and co-expression to be TCONS_00104103, TCONS_00162586, TCONS_00172817, TCONS_00082721, and TCONS_00143117. Interestingly, the lincRNA TCONS_00082721 with *FABP4* as the candidate target gene is also a DELs that regulates milk fat metabolism predicted by the co-localization and co-expression. To clarify this hypothesis, it needs to be further verified in functional experiments. These findings will be of great significance for further research on the mechanism of milk fat synthesis in dairy cows.

### Protein Interaction and LncRNA_miRNA_mRNA Regulatory Network Analysis

As an important regulatory factor, miRNAs can change the expression of target mRNA and involve widely in various biological processes. In recent years, more and more studies have shown that lncRNAs have the function of sponge adsorption of miRNAs and can shield the inhibition or degradation of target genes by competitive binding of miRNAs. And rapidly upregulate its expression (Wang et al., 2018; Zhang and Lu, 2018). In this study, we first predicted the targeted differential miRNAs of DELs and then predicted the target genes of differential miRNAs. Thirty-four target genes related to lipid metabolism of differential miRNAs with DELs targeting relationship were screened out by GO/KEGG enrichment analysis.

Protein interaction network analysis showed that the target genes *HADHA*, *HADHB*, *ACAA1*, *ACADS*, *ACADSB*, and *ACAT1* had a strong interaction relationship and might have common functions. *HADHB* is involved in the β-oxidation of fatty acids and catalyzes the second to fourth steps of the β-oxidation of fatty acid. The other five genes are acyltransferases or dehydrogenases, which play important regulatory roles in the β-oxidation of fatty acids (Fernándezhernando et al., 2011). Also, the interaction between *FASN*, *OXSM*, and *MCAT* genes is strongly supported by the data. *FASN* is an enzyme involved in fatty acid (FA) synthesis and plays a significant role in the formation of new adipogenesis in mammals. It has been found that the variation of *FASN* is related to a variety of dairy traits, including milk fat content, total milk solid content and peak milk yield, etc (Matsumoto et al., 2012); Shi et al. (2019) found that *FASN* variation can be used as a genetic marker to improve milk fat content and milk solid level of yaks. The co-overexpression of *SCAP* + *SREBP1* will also lead to the increase in the abundance of *FASN* gene mRNA and the formation of lipid droplets (Han et al., 2018). In short, the current study on the *FASN* gene in the milk fat synthesis has been relatively mature at present. *OXSM* is a 3-oxyacyl -[acyl carrier protein] synthase. Xiong et al. (2020) found that *OXSM* has inhibitory effects on fat catabolism, fat anabolism, and fatty acid oxidation, and promotes glycerol transport and polyunsaturated fatty acid synthesis. *MCAT* is also an important gene involved in lipid metabolism (Ghadge et al., 2020). Therefore, there is an inevitable interaction between *FASN*, *OXSM*, and *MCAT* genes, and they all play a potential role together in the regulation of lipid metabolism. *MAPK3* plays a major role in autophagy, and there is evidence that *MAPK3* is involved in lipid metabolism (Xiao et al., 2016). We also found that the *MAPK3* gene has a strong interaction with multiple lipid metabolism-related genes (*JUN*, *SMAD2*, *GH1*, *SPHK2*, *FASN*, *PTPRR*, *PLA2G4E*, *PLA2G4F*, *PLA2G4D*, *FGF1*, and *PLCG1*). It is speculated that this gene may play a crucial role in the lipid metabolism process, and the detailed mechanism needs further study.

Through functional analysis, a total of 156 milk fat metabolism-related ceRNAs were constructed in this study. Among the miRNAs targeted by DELs, miR-145 is significantly upregulated in the mammary gland of mid-lactation high milk fat cows (Xia et al., 2021) and has been shown to regulate lipid drop-formation and TAG accumulation in goat mammary epithelial cells (Wang et al., 2017). The regulatory network centered on miR-145 will be the focus of subsequent experimental research. Since TCONS_00082721 is a differential lincRNA predicted by co-localization and co-expression that may regulate milk fat metabolism, the competitive regulatory relationship between TCONS_00082721_miR-145_FASN and TCONS_ 00082721_miR-145_TNF will be further studied and cell experiments will be carried out to verify the interaction and function between them. The target gene predicted by TCONS_00172817 is FABP4 (Dong et al., 2020), which encodes a highly abundant fatty acid-binding protein in the mammary glands of dairy cows. These lncRNAs may regulate milk fat metabolism predicted by the co-localization and co-expression. Therefore, the ceRNAs regulatory network related to TCONS_00172817 is likely to be involved in the process of milk fat synthesis and needs attention. For non-lipid metabolism DELs,TCONS_00045874 and TCONS_00083670 may play a role in miR-145, regulating the expression of lipid metabolism-related genes FASN (Han et al., 2018) and TNF (Alexis et al., 2018). Among all the DELs related to lipid metabolism targeted differential miRNAs, bta-miR-2387 has the most abundant predicted target genes, which may be an important gene in the process of milk fat synthesis in dairy cows. Therefore, the ceRNAs regulatory network with bta-miR-2387 as the core will have more choices in functional verification of its interaction and function, and the possibility of success will also be greater. To sum up, the ceRNAs network analysis was performed on the transcriptome data (mRNA, miRNAs, lncRNAs) obtained from BMECs with high and low MFP. It provides a new way to understand the function of genes in the biological process of milk fat metabolism, and it also provides a new perspective for studying the lactation process of dairy cows.

## Conclusion

In this study, 38 DELs and 47 DEGs were identified in BMECs, all DELs were newly identified. The identification of novel lncRNAs significantly expanded the repertoire of lncRNAs for dairy cows and consequently will help to understand the functions of the bovine lncRNAs. Eighteen lipid metabolism-related DELs and 11 lipid metabolism-related DEGs of were screened by functional enrichment analysis, while 156 ceRNAs interactions regulatory networks related to milk lipid metabolism were constructed, suggesting that ceRNAs may be involved in the lactation process. Our studies provides a new way to understand the gene functions in milk lipid metabolism in ruminant livestock.

## Data Availability

All data generated or analyzed in this study are included in this article [and its Supplementary Material], and the datasets have been submitted to the SRA database with the accession number PRJNA730595. Access to the data of permanent link to https://www.ncbi.nlm.nih. gov/sra/PRJNA730595.

## Abbreviations

BMECs, bovine mammary epithelial cells; BP, biological process; CC, cellular composition; DEGs, differentially expressed genes; DELs, differentially expressed lncRNAs; DHI, dairy herd improvement; FPKM, quantified using fragments per kilobase of exon per millions of reads; GO, gene ontology; KEGG, kyoto encyclopedia of genes and genomes; LncRNAs, long noncoding RNAs; lincRNA, long intervening/intergenic noncoding RNA; miRNAs, microRNAs; MFP, milk fat percentage; MF, molecular function; ncRNAs, non-coding RNAs; Padj, the corrected P-value; RNA-seq, RNA sequencing; qRT-PCR, quantitative real-time reverse transcription PCR; SCC, somatic cell count; TAG, triglyceride.
