# Supplementary material for: Screening and Conjoint Analysis of Key lncRNAs for Milk Fat Metabolism in Dairy Cows
Source: Front Genet. 2022 Feb 3;13:772115. doi: 10.3389/fgene.2022.772115 (PMC8850724; doi:10.3389/fgene.2022.772115)
Supplement: Supplementary file 1 [file Table1.DOCX]

Supplementary Table 1

Lipid metabolism related genes

| Type | Gene | Description |
| --- | --- | --- |
| DEGs | BCAT1 | Branched-chain-aminotransferase-1 |
|  | ENPP2 | Phosphodiesterase family member 2 |
|  | CES4A | Type-B carboxylesterase 4A |
|  | ATP8A2 | ATPase phospholipid transporting 8A2 |
|  | CTSH | Cathepsin H |
|  | APOL3 | Apolipoprotein L3 |
|  | PDGFD | Platelet derived growth factor D |
|  | KCNMA1 | Potassium calcium-activated channel subfamily M alpha 1 |
|  | PTPRR | Protein tyrosine phosphatase receptor type R |
|  | ZFYVE28 | Zinc finger FYVE-type containing 28 |
|  | DKK1 | Dickkopf WNT signaling pathway inhibitor 1 |
| Target genes(cis)  of DELs | SYK | Spleen associated tyrosine kinase |
|  | PLPP2 | Phospholipid phosphatase 2 |
|  | ATP4A | ATPase H+/K+ transporting subunit alpha |
|  | AMPH | Amphiphysin |
|  | VARS2 | Valyl-tRNA synthetase 2 |
|  | ITGA6 | Integrin subunit alpha 6 |
|  | FGF1 | Fibroblast growth factor 1 |
| Target genes(trans)  of DELs | PLA2G4E | Phospholipase A2 group IVE |
|  | FABP4 | Fatty acid binding protein 4 |
|  | OXSM | 3-oxoacyl-ACP synthase |
|  | FABP7 | Fatty acid binding protein 7 |
|  | FABP3 | Fatty acid binding protein 3 |
|  | GPX1 | Glutathione peroxidase 1 |
|  | CYP27A1 | Cytochrome P450 family 27 subfamily A member 1 |
|  | INPP4B | Inositol polyphosphate-4-phosphatase type II B |
|  | ACADSB | Acyl-CoA dehydrogenase short/branched chain |
|  | ALOX12 | Arachidonate 12-lipoxygenase, 12S type |
| Target genes related to Lipid Metabolism of differential miRNA targeted by DELs | HADHA | Hydroxyacyl-CoA dehydrogenase trifunctional multienzyme complex subunit alpha |
|  | TEAD2 | TEA domain transcription factor 2 |
|  | TBL1X | Transducin beta like 1 X-linked |
|  | PLA2G3 | Phospholipase A2 group III |
|  | FASN | Fatty acid synthase |
|  | TNF | Tumor necrosis factor |
|  | ACAA1 | Acetyl-CoA acyltransferase 1 |
|  | LEP | Leptin |
|  | SPHK2 | Sphingosine kinase 2 |
|  | PLTP | Phospholipid transfer protein |
|  | PLA2G4D | Phospholipase A2 group IVD |
|  | CYP26B1 | Cytochrome P450 family 26 subfamily B member 1 |
|  | PLCG1 | Phospholipase C gamma 1 |
|  | CEL | Carboxyl ester lipase |
|  | HADHB | Hydroxyacyl-CoA dehydrogenase trifunctional multienzyme complex subunit beta |
|  | SMAD2 | SMAD family member 2 |
|  | MLYCD | Malonyl-CoA decarboxylase |
|  | PLA2G4F | Phospholipase A2 group IVF |
|  | RXRA | Retinoid X receptor alpha |
|  | PLA2G15 | Phospholipase A2 group XV |
|  | MAPK3 | Mitogen-activated protein kinase 3 |
|  | ACADS | Acyl-CoA dehydrogenase short chain |
|  | MCAT | Malonyl-CoA-acyl carrier protein transacylase |
|  | GH1 | Growth hormone 1 |
|  | SOCS5 | Suppressor of cytokine signaling 5 |
|  | JUN | Jun proto-oncogene, AP-1 transcription factor subunit |
|  | PPP2R5A | Protein phosphatase 2 regulatory subunit B'alpha |
|  | APOC3 | Apolipoprotein C3 |
|  | PNPLA2 | Patatin like phospholipase domain containing 2 |
|  | FADS2 | Fatty acid desaturase 2 |
|  | PLA2G2A | Phospholipase A2 group IIA |
|  | MTM1 | Myotubularin 1 |
|  | LBP | Lipopolysaccharide binding protein |
|  | DIS3L2 | DIS3 like 3'-5' exoribonuclease 2 |
|  | NDRG1 | N-myc downstream regulated 1 |
|  | SRRM2 | Serine/arginine repetitive matrix 2 |
|  | ANO1 | Anoctamin 1 |
|  | ACAT1 | Acetyl-CoA acetyltransferase 1 |
| Other lipid metabolism related genes | LPL | Lipoprotein lipase |
|  | GPAM | Glycerol-3-phosphate acyltransferase, mitochondrial |
|  | MSMO1 | Methylsterol monooxygenase 1 |
|  | SCD | Stearoyl-CoA desaturase |
|  | PPAR γ | Peroxisome proliferator activated receptor gamma |

Supplementary Table 2

Primer sequence and annealing temperature

| GenBank ID | Genes/DELs | Primer sequence(5’-3’) | Product length /bp | Annealing temperature /℃ |
| --- | --- | --- | --- | --- |
| NM_001033610.1 | Keratin 8 F | ATTAACAACCTCCGTCGGCA | 142 | 60.0 |
|  | Keratin 8 R | TCTCCATGTCTGTGCGCTTT |  |  |
| NM_181024.2 | PPARγ F | TGCCTTTGACATCAAGCCCT | 154 | 60.0 |
|  | PPARγ R | CTCCACTTTGATTGCACTTTGGT |  |  |
| NM_174313.2 | FABP3 F | CAGGCAGGTGGGCAATATGA | 170 | 60.0 |
|  | FABP3 R | GCGTCACGATGGACTTGACTT |  |  |
| NM_001012669.1 | FASN F | AGACGGTGCTCATTCACTCG | 106 | 60.0 |
|  | FASN R | TTTTCGGCTGACCCCACAAG |  |  |
| NM_173959.4 | SCD F | CATGGCGTTCCAGAATGACG | 108 | 60.0 |
|  | SCD R | AAGAAAAAGCCACGTCGGGA |  |  |
| NM_001034034.2 | GAPDH F | TCGGAGTGAACGGATTCGG | 192 | 60.0 |
|  | GAPDH R | TGATGACGAGCTTCCCGTTC |  |  |
| NM_001167834.1 | CES4A F | TTTGAGCACTACGCTCCTGG | 188 | 58.2 |
|  | CES4A R | CCACCATTGGGGTTTCCTGT |  |  |
| NM_001163802.3 | ATP8A2 F | GCCCACAGCTGGAGAAGATA | 189 | 59.4 |
|  | ATP8A2 R | GTACTTGGCCGTGCTGATCT |  |  |
| NM_001034385.2 | CTSH F | CGCCCAGAACTTCAACAACC | 132 | 59.4 |
|  | CTSH R | TACTTGCAGTCACCATCCTGG |  |  |
| NM_001100297.1 | APOL3 F | GCAGACTCCTGGGGTGAAAC | 247 | 58.2 |
|  | APOL3 R | TGGACAAATCAGCCTCGGTC |  |  |
| NM_001205544.1 | DKK1 F | ATTGACAACCACCAGCCGTA | 189 | 59.4 |
|  | DKK1 R | AGAAGGCATGCATATCCCGT |  |  |
| NM_001080293.1 | ENPP2 F | TCACTTTTGCCGTCGGTGTCAA | 174 | 58.0 |
|  | ENPP2 R | AATCAGGGGGTCCAGCCTCTTG |  |  |
| NM_001083644.1 | BCAT1 F | TGTGTTGTTTGCCCTGTTTC | 138 | 59.5 |
|  | BCAT1 R | GTCGCTCTCTTCTCTTCCGT |  |  |
| NM_001113261.1 | PTPRR F | TGAGGACAAGACAGCCAACAG | 129 | 56.4 |
|  | PTPRR R | AAAGGAGAAGGGCAGACAGAG |  |  |
|  | TCONS_00172817 F | CCAAACACTGGCCATGAAGAA | 233 | 60.9 |
|  | TCONS_00172817 R | GCTCGGTAAGCAGCAAGAAGA |  |  |
|  | TCONS_00143115 F | TATCTTGGTTGGCCTCATTGATT | 169 | 56.4 |
|  | TCONS_00143115 R | CATGTGGGTCTCTGTGACTTCTG |  |  |
|  | TCONS_00191498 F | CAGTTCTCAGTGTTGCTTCTTG | 198 | 56.4 |
|  | TCONS_00191498 R | TTACATCTGTCCTGCTTTCATG |  |  |
|  | TCONS_00007612 F | AGCGAAGCAAGTTTCCTACA | 196 | 59.5 |
|  | TCONS_00007612 R | AACACAATCACTGGCCCATA |  |  |
|  | TCONS_00143117 F | GTCTTCTACTTCAGCCTCAACC | 198 | 60.9 |
|  | TCONS_00143117 R | GACCTCAAACTGCTCTGTCATT |  |  |
|  | TCONS_00119434 F | ATGGGGTCACGAATGAGTCAGA | 166 | 57.8 |
|  | TCONS_00119434 R | GCTAGAGGCCAGGATCAAGAGG |  |  |
|  | TCONS_00082721 F | CCTCCTCCTTCTCTTCTTCTGCC | 163 | 60.9 |
|  | TCONS_00082721 R | CTTCAAGTGACCTTCCTCTCCCC |  |  |
|  | TCONS_00054231 F | ATGAGAAGGTGAAAATTGGGGAG | 105 | 60.9 |
|  | TCONS_00054231 R | TTTGATGAGCAAGAGCAGAGACT |  |  |

DELs = Differentially expressed lncRNAs

Supplementary Table 3

GO/KEGG enrichment of DEGs related to lipid metabolism

| Genes | Functional annotation | | | | Pathway | *P*-value |
| --- | --- | --- | --- | --- | --- | --- |
|  | Biological process | *P*-value | Molecular function | *P*-value |  |  |
| BCAT1 | Carboxylic acid biosynthetic process | 0.3906 | Transaminase activity | 0.0401 | Pantothenate and CoA biosynthesis | 0.0401 |
|  |  |  |  |  | 2-Oxocarboxylic acid metabolism | 0.0401 |
| ENPP2 | Glycerophospholipid catabolic process | 0.0375 | Alkylglycerophosphoethanolamine phosphodiesterase activity | 0.0041 | Ether lipid metabolism | 0.0880 |
|  |  |  | Phosphodiesterase I activity | 0.0135 |  |  |
|  |  |  | Carboxylic ester hydrolase activity | 0.0210 |  |  |
|  |  |  | Lysophospholipase activity | 0.0257 |  |  |
|  |  |  | Lipase activity | 0.0292 |  |  |
| CES4A | Lipid catabolic process | 0.1108 | Sterol esterase activity | 0.0147 |  |  |
|  |  |  | Carboxylic ester hydrolase activity | 0.0210 |  |  |
|  |  |  | Hydrolase activity | 0.0261 |  |  |
|  |  |  | Lipase activity | 0.0292 |  |  |
|  |  |  | Triglyceride lipase activity | 0.0385 |  |  |
| ATP8A2 | Regulation of phospholipid translocation | 0.0227 | Aminophospholipid transporter activity | 0.0214 |  |  |
|  | Positive regulation of phospholipid translocation | 0.0227 |  |  |  |  |
|  | Positive regulation of phospholipid transport | 0.0238 | Hydrolase activity | 0.0261 |  |  |
|  | Regulation of phospholipid transport | 0.0255 |  |  |  |  |
| CTSH | Response to lipid | 0.6564 | Hydrolase activity | 0.0261 | Lysosome | 0.2246 |
| APOL3 | Lipid transport | 0.1289 | Lipid binding | 0.4894 |  |  |
| PDGFD | Inositol lipid-mediated signaling | 0.3669 | Platelet-derived growth factor receptor binding | 0.0329 | Rap1 signaling pathway | 0.0099 |
|  |  |  |  |  | Ras signaling pathway | 0.0840 |
|  |  |  |  |  | PI3K-Akt signaling pathway | 0.5109 |
| KCNMA1 | Lipid metabolic process | 0.3996 | Transporter activity | 0.3934 | cGMP-PKG signaling pathway | 0.2918 |
| PTPRR | Regulation of epithelial cell migration | 0.0140 | Hydrolase activity | 0.0261 | MAPK signaling pathway | 0.4143 |
| ZFYVE28 | Regulation of receptor activity | 0.0024 | Lipid binding | 0.48948 |  |  |
| DKK1 | Regulation of canonical Wnt receptor signaling pathway | 0.0040 | Receptor binding | 0.1400 | Wnt signaling pathway | 0.0333 |

DEGs = differentially expressed genes.

Supplementary Table 4

GO/KEGG enrichment of target genes related to lipid metabolism in DELs(co-localization)

| Genes | Functional annotation | | | | Pathway | *P*-value |
| --- | --- | --- | --- | --- | --- | --- |
|  | Biological process | *P*-value | Molecular function | *P*-value |  |  |
| SYK | Regulation of arachidonic acid secretion  Regulation of fatty acid transport | 0.0138  0.0582 | Phosphatase binding | 0.1419 | Fc gamma R-mediated phagocytosis | 0.0077 |
|  |  |  |  |  | PI3K-Akt signaling pathway | 0.2105 |
|  |  |  |  |  | NF-kappa B signaling pathway | 0.3388 |
| PPAP2C | Phospholipid dephosphorylation | 0.1472 | Lipid phosphatase activity | 0.0375 | Fc gamma R-mediated phagocytosis | 0.0077 |
|  |  |  |  |  | Ether lipid metabolism | 0.1848 |
|  |  |  |  |  | Glycerolipid metabolism | 0.2316 |
| VARS2 | Amino acid activation | 0.2112 | Carboxylic ester hydrolase activity | 0.4603 | Aminoacyl-tRNA biosynthesis | 0.2691 |
| ITGA6 | Brown fat cell differentiation | 0.1730 | Insulin-like growth factor I binding | 0.05419 | PI3K-Akt signaling pathway | 0.2105 |
| FGF1 | Regulation of lipid biosynthetic process | 0.2417 | Fibroblast growth factor receptor binding | 0.1312 | PI3K-Akt signaling pathway | 0.2105 |
|  |  |  |  |  | Rap1 signaling pathway | 0.2561 |
|  |  |  |  |  | Hippo signaling pathway | 0.4994 |
|  |  |  |  |  | MAPK signaling pathway | 0.6946 |
| ATP4A | Transmembrane transport | 0.3719 | Hydrolase activity, acting on acid anhydrides | 0.5045 | Oxidative phosphorylation | 0.0287 |
| AMPH | Establishment of organelle localization | 0.1994 | Phospholipid binding | 0.5348 | Fc gamma R-mediated phagocytosis | 0.0077 |

DELs = Differentially expressed lncRNAs.

Supplementary Table 5

GO/KEGG enrichment of target genes related to lipid metabolism in DELs(co-expression)

| Genes | Functional annotation | | | | Pathway | *P*-value |
| --- | --- | --- | --- | --- | --- | --- |
|  | Biological brocess | *P*-value | Molecular function | *P*-value |  |  |
| PLA2G4E | Lipid metabolic process  Cellular lipid metabolic process | 0.0089  0.0174 | Lipid binding | 0.0405 | Fc epsilon RI signaling pathway | 0.0329 |
|  |  |  |  |  | GnRH signaling pathway | 0.0377 |
|  |  |  |  |  | Arachidonic acid metabolism | 0.0483 |
| FABP4 | Lipid metabolic process | 0.0089 | Fatty acid transporter activity | 0.0060 | PPAR signaling pathway | 0.0152 |
|  | Cellular lipid metabolic process | 0.0174 | Carboxylic acid binding | 0.0079 |  |  |
|  | Fatty acid transport | 0.0178 | Long-chain fatty acid binding | 0.0096 |  |  |
|  | Long-chain fatty acid transport | 0.0181 | Fatty acid binding | 0.0132 |  |  |
|  | Fatty acid metabolic process | 0.0447 | Long-chain fatty acid transporter activity | 0.0165 |  |  |
| OXSM | Lipid metabolic process | 0.0089 | Fatty acid synthase activity | 0.0035 | Fatty acid metabolism | 0.0291 |
|  | Cellular lipid metabolic process | 0.0174 |  |  |  |  |
|  | Fatty acid biosynthetic process | 0.0187 |  |  |  |  |
|  | Fatty acid metabolic process | 0.0447 |  |  |  |  |
| GPX1 | Lipid metabolic process | 0.0089 | Oxidoreductase activity | 0.0449 | Glutathione metabolism | 0.0001 |
|  | Cellular lipid metabolic process | 0.0174 |  |  | Arachidonic acid metabolism | 0.0483 |
| CYP27A1 | Lipid metabolic process | 0.0089 | Cholesterol 26-hydroxylase activity | 0.0302 | PPAR signaling pathway | 0.0152 |
| INPP4B | Lipid metabolic process | 0.0089 | Phosphatidylinositol-3,4-bisphosphate 4-phosphatase activity | 0.0691 | Phosphatidylinositol signaling system | 0.0706 |
|  | Cellular lipid metabolic process | 0.0174 |  |  |  |  |
| ACADSB | Lipid metabolic process | 0.0089 | Oxidoreductase activity | 0.0449 | Fatty acid metabolism | 0.0291 |
|  | Cellular lipid metabolic process | 0.0174 |  |  |  |  |
|  | Fatty acid metabolic process | 0.0447 |  |  |  |  |
| FABP7 | Cell differentiation | 0.0638 | Lipid binding | 0.0405 | PPAR signaling pathway | 0.0152 |
| FABP3 |  |  | Lipid binding | 0.0405 | PPAR signaling pathway | 0.0152 |
| ALOX12 | Unsaturated fatty acid biosynthetic process | 0.0016 | Linoleate 13S-lipoxygenase activity | 0.0587 | Arachidonic acid metabolism | 0.0483 |
|  | Lipid metabolic process | 0.0089 |  |  |  |  |
|  | Fatty acid biosynthetic process | 0.0187 |  |  |  |  |

DELs = Differentially expressed lncRNAs.

Supplementary Table 6

DELs and target genes related to lipid metabolism(co-localization)

| DELs ID | DELs location | Gene name | Gene start | Gene end | Distance | Location |
| --- | --- | --- | --- | --- | --- | --- |
| TCONS_00104103 | 23:27943191-28058062 | VARS2 | 28146080 | 28159087 | 88018 | downstream |
| TCONS_00082721 | 2:24262990-24300737 | ITGA6 | 24091576 | 24179151 | 83838 | upstream |
| TCONS_00066920 | 18:46165575-46169765 | ATP4A | 46209079 | 46222026 | 39314 | downstream |
| TCONS_00162586 | 7:42726724-42986751 | PLPP2 | 43016867 | 43028554 | 30116 | downstream |
| TCONS_00163391 | 7:53750360-53759144 | FGF1 | 53622272 | 53729308 | 21051 | upstream |
| TCONS_00143115 | 4:82475604-82533958 | AMPH | 82295678 | 82507550 | -31947 | antisense |
| TCONS_00172817 | 8:86876741-87001036 | SYK | 86977448 | 87083681 | -23588 | antisense |
| TCONS_00143117 | 4:82502392-82533931 | AMPH | 82295678 | 82507550 | -5159 | antisense |

DELs = differentially expressed lncRNAs, DELs location = the specific location of the DELs on the chromosome.

gene start = starting position of DELs target gene, gene end = termination position of DELs target gene, distance = distance between DELs and target gene, location = the positional relationship between DELs and the target gene.

Supplementary Table 7

DELs and target genes related to lipid metabolism(co-expression)( R^2^>0.97)

| DELs ID | Gene name | Pearson correlation(R^2^) | *P*-value |
| --- | --- | --- | --- |
| TCONS_00104103 | ACADSB | 0.9950 | 2.97E-07 |
| TCONS_00058915 | PLA2G4E | 0.9875 | 4.79E-06 |
| TCONS_00143117 | FABP7 | 0.9823 | 1.36E-05 |
| TCONS_00054231 | INPP4B | 0.9799 | 1.98E-05 |
| TCONS_00162586 | INPP4B | 0.9798 | 2.02E-05 |
| TCONS_00191498 | CYP27A1 | 0.9797 | 2.04E-05 |
| TCONS_00162586 | ACADSB | 0.9794 | 2.15E-05 |
| TCONS_00082721 | FABP4 | 0.9788 | 2.33E-05 |
| TCONS_00164841 | INPP4B | 0.9784 | 2.45E-05 |
| TCONS_00150126 | GPX1 | 0.9782 | 2.52E-05 |
| TCONS_00104103 | FABP3 | 0.9761 | 3.35E-05 |
| TCONS_00054231 | ACADSB | 0.9754 | 3.62E-05 |
| TCONS_00172817 | FABP4 | 0.9754 | 3.62E-05 |
| TCONS_00007612 | OXSM | 0.9746 | 3.99E-05 |
| TCONS_00164841 | ACADSB | 0.9744 | 4.11E-05 |
| TCONS_00119434 | ALOX12 | 0.9733 | 4.65E-05 |
| TCONS_00027906 | ACADSB | 0.9726 | 5.00E-05 |
| TCONS_00118412 | CYP27A1 | 0.9716 | 5.59E-05 |
| TCONS_00156313 | PLA2G4E | 0.9716 | 5.57E-05 |
| TCONS_00156313 | INPP4B | 0.9703 | 6.40E-05 |

Supplementary Table 8

Differential miRNAs

| sRNA | HMF_readcount | LMF_readcount | log2FoldChange | pval | padj |
| --- | --- | --- | --- | --- | --- |
| bta-miR-19a | 33.6902836 | 5.86836971 | 2.0333 | 2.39E-06 | 0.00054797 |
| bta-miR-450a | 12.71314398 | 152.3315576 | -1.9747 | 0.00034071 | 0.039011 |
| bta-miR-182 | 2277.235944 | 5234.320597 | -1.0929 | 0.00060721 | 0.046351 |
| bta-miR-18a | 70.36371942 | 22.04539449 | 1.3679 | 0.001159 | 0.066352 |
| bta-miR-197 | 155.6685881 | 303.7130979 | -0.89263 | 0.0019632 | 0.07249 |
| bta-miR-455-3p | 39.86308433 | 87.07670405 | -1.0116 | 0.0021527 | 0.07249 |
| bta-miR-1343-3p | 71.03429206 | 178.4706666 | -1.1508 | 0.0022158 | 0.07249 |
| bta-miR-2285s | 23.47270309 | 5.872291136 | 1.4529 | 0.0037894 | 0.096432 |
| bta-miR-106b | 171.8727108 | 97.98201055 | 0.75967 | 0.0041956 | 0.096432 |
| bta-miR-2285f | 601.0185611 | 225.1642873 | 1.1846 | 0.004211 | 0.096432 |
| bta-miR-2299-5p | 14.16134007 | 3.414770231 | 1.3999 | 0.005977 | NA |
| bta-miR-331-3p | 33.38214956 | 63.1528906 | -0.8302 | 0.007072 | 0.13949 |
| bta-miR-20a | 1218.490855 | 576.347833 | 0.95127 | 0.0082192 | 0.13949 |
| bta-miR-2387 | 58.39276826 | 128.3725886 | -0.98597 | 0.0084998 | 0.13949 |
| bta-miR-222 | 2967.302205 | 5909.16749 | -0.89173 | 0.0085275 | 0.13949 |
| bta-miR-2285l | 5.014047639 | 26.00184116 | -1.4183 | 0.009238 | 0.14036 |
| bta-miR-2285ce | 94.37787353 | 53.78788085 | 0.75391 | 0.0098329 | 0.14036 |
| bta-miR-106a | 4.54871945 | 0.409682182 | 1.4362 | 0.01005 | NA |
| bta-miR-183 | 1514.369385 | 3677.565353 | -1.066 | 0.01042 | 0.14036 |
| bta-miR-99a-5p | 11793.45354 | 17676.68476 | -0.55945 | 0.012382 | 0.15752 |
| bta-miR-375 | 19.67187798 | 39.30595649 | -0.86226 | 0.013472 | 0.16237 |
| bta-miR-2285bn | 94.16497638 | 54.84714319 | 0.72108 | 0.015363 | 0.16809 |
| bta-miR-22-5p | 118.7169315 | 190.5891125 | -0.63739 | 0.015415 | 0.16809 |
| bta-miR-2285cb | 2.324352029 | 0 | 1.2474 | 0.018878 | NA |
| bta-miR-421 | 43.77675998 | 70.27205451 | -0.63813 | 0.020298 | 0.21129 |
| bta-miR-221 | 3073.358906 | 5473.918576 | -0.7536 | 0.021383 | 0.2129 |
| bta-miR-6120-3p | 537.8359756 | 347.1570461 | 0.59262 | 0.025461 | 0.24294 |
| bta-miR-2285bs | 6.159463042 | 1.454387387 | 1.2047 | 0.025966 | NA |
| bta-miR-96 | 571.6255977 | 1001.206616 | -0.72749 | 0.028885 | 0.25452 |
| bta-miR-2285k | 54.22493686 | 25.98248675 | 0.87824 | 0.028897 | 0.25452 |
| bta-miR-145 | 10.56258478 | 36.03445683 | -1.1374 | 0.034485 | 0.29249 |
| bta-miR-370 | 10.20944742 | 3.178099568 | 1.101 | 0.037319 | NA |
| novel_330 | 3.646172706 | 0.402403612 | 1.1494 | 0.037798 | NA |
| bta-miR-224 | 339.0414118 | 1373.609857 | -1.0929 | 0.049005 | 0.37566 |

Supplementary Table 9

LncRNAs that may be precursors of miRNA

| Hairpin ID | Hairpin start | Hairpin end | LncRNA ID | LncRNA start | LncRNA end |
| --- | --- | --- | --- | --- | --- |
| bta-miR-11986 | 4 | 54 | TCONS_00007954 | 5619 | 5668 |
| bta-miR-11986 | 4 | 54 | TCONS_00007955 | 5710 | 5759 |
| bta-miR-11986 | 4 | 54 | TCONS_00031230 | 1876 | 1926 |
| bta-miR-11986 | 4 | 54 | TCONS_00041829 | 3500 | 3550 |
| bta-miR-125b-1 | 1 | 88 | TCONS_00047662 | 4797 | 4884 |
| bta-miR-125b-1 | 1 | 88 | TCONS_00047663 | 4797 | 4884 |
| bta-miR-11986 | 4 | 54 | TCONS_00047751 | 830 | 880 |
| bta-miR-11986 | 4 | 54 | TCONS_00047752 | 723 | 773 |
| novel_181 | 1 | 85 | TCONS_00061186 | 2688 | 2772 |
| bta-miR-423 | 1 | 94 | TCONS_00069938 | 13 | 106 |
| novel_305 | 1 | 53 | TCONS_00073201 | 3848 | 3902 |
| novel_305 | 1 | 53 | TCONS_00073202 | 4150 | 4204 |
| novel_305 | 1 | 53 | TCONS_00073203 | 3845 | 3899 |
| novel_305 | 1 | 53 | TCONS_00073206 | 4751 | 4805 |
| novel_305 | 1 | 53 | TCONS_00073207 | 4295 | 4349 |
| bta-miR-11978 | 1 | 60 | TCONS_00083004 | 1199 | 1258 |
| bta-mir-11978 | 1 | 60 | TCONS_00083005 | 2677 | 2736 |
| novel_97 | 1 | 69 | TCONS_00092871 | 562 | 630 |
| novel_97 | 1 | 69 | TCONS_00092872 | 511 | 579 |
| bta-miR-11986 | 6 | 54 | TCONS_00127674 | 365 | 414 |
| bta-miR-11986 | 4 | 54 | TCONS_00138211 | 4628 | 4678 |
| bta-miR-11986 | 4 | 54 | TCONS_00140772 | 240 | 290 |
| bta-miR-11986 | 4 | 54 | TCONS_00153102 | 1170 | 1220 |
| novel_349 | 1 | 57 | TCONS_00156965 | 68 | 124 |
| bta-miR-11986 | 4 | 54 | ENSBTAT00000075954 | 657 | 707 |
| bta-miR-22 | 1 | 85 | ENSBTAT00000084074 | 713 | 797 |
| bta-miR-11986 | 4 | 54 | ENSBTAT00000067952 | 210 | 260 |
| bta-miR-101-1 | 1 | 83 | ENSBTAT00000081296 | 354 | 436 |
| bta-miR-11986 | 4 | 54 | ENSBTAT00000075183 | 2150 | 2200 |
| bta-miR-11986c | 1 | 60 | ENSBTAT00000072445 | 122 | 181 |

Hairpin id = ID of miRNA precursor, hairpin start = the starting position of the homologous part of miRNA precursor and lncRNA on the miRNA precursor, hairpin end = the termination position of the homologous part of miRNA precursor and lncRNA on the miRNA precursor, lncRNA start = the starting position of the homologous part of lncRNA and miRNA precursor on lncRNA, lncRNA end = the termination position of the homologous part of lncRNA and miRNA precursor on lncRNA.

Supplementary Table 10

GO/KEGG enrichment of target genes related to lipid metabolism of targeted differential miRNA of DELs

| Genes | Functional annotation | | | | Pathway | *P*-value |
| --- | --- | --- | --- | --- | --- | --- |
|  | Biological process | *P*-value | Molecular function | *P*-value |  |  |
| PLA2G3 | Cellular lipid metabolic process | 0.0004 | Hydrolase activity, acting on ester bonds | 0.0415 | Ras signaling pathway | 0.0087 |
|  | Glycerolipid metabolic process | 0.0025 | Carboxylic ester hydrolase activity | 0.0500 | alpha-Linolenic acid metabolism | 0.0514 |
|  | Glycerophospholipid metabolic process | 0.0051 | Lipase activity | 0.0584 | Glycerophospholipid metabolism | 0.0571 |
| SPHK2 | Cellular lipid metabolic process | 0.0004 | Phosphotransferase activity, alcohol group as acceptor | 0.0002 | Calcium signaling pathway | 0.0733 |
|  | Lipid biosynthetic process | 0.0007 | Kinase activity | 0.0005 | Fc gamma R-mediated phagocytosis | 0.1603 |
|  | Phospholipid metabolic process | 0.0008 | Bioactive lipid receptor activity | 0.0266 | Sphingolipid metabolism | 0.5038 |
| PLTP | Lipid metabolic process | 0.0016 | Phospholipid binding | 0.0317 | PPAR signaling pathway | 0.1562 |
|  |  |  | Phosphatidylethanolamine binding | 0.0318 |  |  |
| PLA2G4D | Cellular lipid metabolic process | 0.0004 | Phospholipid binding | 0.0317 | MAPK signaling pathway | 0.0048 |
|  | Glycerolipid metabolic process | 0.0025 | Hydrolase activity, acting on ester bonds | 0.0415 | Ras signaling pathway | 0.0087 |
|  | Glycerophospholipid metabolic process | 0.0051 | Carboxylic ester hydrolase activity | 0.0500 | Glycerophospholipid metabolism | 0.0571 |
| CYP26B1 | Cellular lipid metabolic process | 0.0004 | Lipid binding | 0.0527 | Metabolic pathways | 0.4170 |
|  | Response to lipid | 0.0357 |  |  |  |  |
|  | Cellular lipid catabolic process | 0.0365 |  |  |  |  |
| PLCG1 | Cellular lipid metabolic process | 0.0004 | Hydrolase activity, acting on ester bonds  Lipase activity | 0.0415  0.0584 | Ras signaling pathway | 0.0087 |
|  | Phospholipid metabolic process | 0.0008 |  |  | Rap1 signaling pathway | 0.0090 |
|  | Cellular lipid catabolic process | 0.0365 |  |  | NF-kappa B signaling pathway | 0.0099 |
| CEL | Cellular lipid metabolic process  Cellular lipid catabolic process | 0.0004  0.0365 | Hydrolase activity, acting on ester bonds | 0.0415 | Glycerolipid metabolism | 0.0952 |
|  |  |  | Carboxylic ester hydrolase activity | 0.0500 | Steroid biosynthesis | 0.2630 |
|  |  |  | Lipase activity | 0.0584 | Fat digestion and absorption | 0.2832 |
| PLA2G4F | Cellular lipid metabolic process | 0.0004 | Hydrolase activity, acting on ester bonds | 0.0415 | MAPK signaling pathway | 0.0048 |
|  | Lipid biosynthetic process | 0.0007 | Carboxylic ester hydrolase activity | 0.0500 | Ras signaling pathway | 0.0087 |
|  | Glycerolipid metabolic process | 0.0025 | Lipid binding | 0.0527 | Glycerophospholipid metabolism | 0.0571 |
| RXRA | Response to lipid | 0.0357 | Lipid binding | 0.0527 | PPAR signaling pathway | 0.1562 |
| PLA2G15 | Cellular lipid metabolic process | 0.0004 | Hydrolase activity, acting on ester bonds | 0.0415 | Glycerophospholipid metabolism | 0.0571 |
|  | Glycerolipid metabolic process | 0.0025 | Carboxylic ester hydrolase activity | 0.0500 |  |  |
|  | Glycerolipid catabolic process | 0.0114 | Lipase activity | 0.0584 |  |  |
| APOC3 | Cellular lipid metabolic process | 0.0004 | Phospholipid binding  Lipid binding | 0.0317  0.0527 | PPAR signaling pathway | 0.1562 |
|  | Lipid biosynthetic process | 0.0007 |  |  |  |  |
|  | Glycerolipid metabolic process | 0.0025 |  |  |  |  |
| PNPLA2 | Cellular lipid metabolic process | 0.0004 | Hydrolase activity, acting on ester bonds | 0.0415 | Glycerolipid metabolism | 0.0952 |
|  | Lipid biosynthetic process | 0.0007 | Carboxylic ester hydrolase activity | 0.0500 |  |  |
|  | Glycerolipid metabolic process | 0.0025 | Lipase activity | 0.0584 |  |  |
| PLA2G2A | Cellular lipid metabolic process | 0.0004 | Phospholipid binding | 0.0317 | Ras signaling pathway | 0.0087 |
|  | Phospholipid metabolic process | 0.0008 | Hydrolase activity, acting on ester bonds | 0.0415 | alpha-Linolenic acid metabolism | 0.0514 |
|  | Lipid catabolic process | 0.0474 | Carboxylic ester hydrolase activity | 0.0500 | Glycerophospholipid metabolism | 0.0571 |
| LBP | Response to lipid | 0.0357 | Lipid binding | 0.0527 | NF-kappa B signaling pathway | 0.0099 |
| HADHA | Cellular lipid metabolic process | 0.0004 | Long-chain-3-hydroxyacyl-CoA dehydrogenase activity | 0.0141 | Fatty acid metabolism | 0.1271 |
|  | Lipid modification | 0.0263 | Carbon-oxygen lyase activity | 0.0174 | Fatty acid degradation | 0.3890 |
|  | Cellular lipid catabolic process | 0.0365 | Hydro-lyase activity | 0.0343 | Fatty acid elongation | 0.7532 |
| TEAD2 | Response to lipid | 0.0357 | Transcription regulatory region DNA binding | 0.0016 | Hippo signaling pathway | 0.6843 |
| TBL1X | Response to lipid | 0.0357 | Transcription regulatory region DNA binding | 0.0016 | Wnt signaling pathway | 0.7331 |
| FASN | Cellular lipid metabolic process | 0.0004 | Hydrolase activity, acting on ester bonds | 0.0415 | Fatty acid metabolism | 0.1271 |
|  | Lipid biosynthetic process | 0.0016 | Fatty acid synthase activity | 0.3418 | Fatty acid biosynthesis | 0.2312 |
| TNF | Cellular lipid metabolic process | 0.0004 | Transcription regulatory region DNA binding | 0.0016 | TNF signaling pathway | 0.0033 |
|  | Lipid biosynthetic process | 0.0007 |  |  | MAPK signaling pathway | 0.0048 |
|  | Acylglycerol metabolic process | 0.0488 |  |  | NF-kappa B signaling pathway | 0.0099 |
| ACAA1 | Cellular lipid metabolic process | 0.0004 | Transferase activity, transferring acyl groups | 0.0327 | alpha-Linolenic acid metabolism | 0.0514 |
|  | Lipid modification | 0.0263 |  |  | Peroxisome | 0.0692 |
|  | Cellular lipid catabolic process | 0.0365 |  |  | PPAR signaling pathway | 0.1562 |
| LEP | Cellular lipid metabolic process | 0.0004 | Receptor binding | 0.0056 | Adipocytokine signaling pathway | 0.1562 |
|  | Lipid biosynthetic process | 0.0007 |  |  | Jak-STAT signaling pathway | 0.2877 |
|  | Cellular lipid catabolic process | 0.0365 |  |  | AMPK signaling pathway | 0.9054 |
| HADHB | Cellular lipid metabolic process | 0.0004 | Acetyl-CoA C-acyltransferase activity | 0.0139 | Fatty acid metabolism | 0.1271 |
|  | Lipid modification | 0.0263 | Long-chain-3-hydroxyacyl-CoA dehydrogenase activity | 0.0141 | Fatty acid degradation | 0.3890 |
|  | Cellular lipid catabolic process | 0.0365 | Lyase activity | 0.0168 | Fatty acid elongation | 0.7532 |
| SMAD2 | Response to lipid | 0.0357 | Transcription regulatory region sequence-specific DNA binding | 0.0139 | Hippo signaling pathway | 0.6843 |
| MLYCD | Cellular lipid metabolic process | 0.0004 | Binding | 0.0006 | Peroxisome | 0.0692 |
|  | Lipid biosynthetic process | 0.0007 | Lyase activity | 0.0168 | Metabolic pathways | 0.4170 |
|  | Lipid modification | 0.0263 | Malonyl-CoA decarboxylase activity | 0.1187 | AMPK signaling pathway | 0.9054 |
| MAPK3 | Response to lipid | 0.0357 | Phosphotransferase activity, alcohol group as acceptor | 0.0002 | TNF signaling pathway | 0.0033 |
|  |  |  |  |  | MAPK signaling pathway | 0.0048 |
|  |  |  |  |  | Ras signaling pathway | 0.0087 |
| ACADS | Cellular lipid metabolic process | 0.0004 | Acyl-CoA dehydrogenase activity | 0.1129 | Fatty acid metabolism | 0.1271 |
|  | Lipid modification | 0.0263 |  |  | Fatty acid degradation | 0.3890 |
|  | Cellular lipid catabolic process | 0.0365 |  |  | Metabolic pathways | 0.4170 |
| MCAT | Cellular lipid metabolic process | 0.0004 | Transferase activity, transferring acyl groups  Fatty acid synthase activity | 0.0327  0.3418 | Fatty acid metabolism | 0.1271 |
|  | Lipid biosynthetic process | 0.0007 |  |  | Fatty acid biosynthesis | 0.2312 |
|  | Lipid metabolic process | 0.0016 |  |  | Metabolic pathways | 0.4170 |
| GH1 | Cellular lipid metabolic process | 0.0004 | Binding | 0.0006 | Jak-STAT signaling pathway  PI3K-Akt signaling pathway | 0.2877  0.5537 |
|  | Glycerolipid metabolic process | 0.0025 |  |  |  |  |
|  | Regulation of diacylglycerol biosynthetic process | 0.0476 |  |  |  |  |
| SOCS5 | Cellular lipid metabolic process | 0.0004 | Phosphatidylinositol 3-kinase regulator activity | 0.4572 | Jak-STAT signaling pathway  Prolactin signaling pathway | 0.2877  0.5236 |
|  | Glycerolipid metabolic process | 0.0025 |  |  |  |  |
|  | Lipid modification | 0.0263 |  |  |  |  |
| JUN | Response to lipid | 0.0357 | Transcription regulatory region DNA binding | 0.0016 | TNF signaling pathway | 0.0033 |
|  |  |  |  |  | MAPK signaling pathway | 0.0048 |
| PPP2R5A | Lipid metabolic process | 0.0016 | Phosphoric ester hydrolase activity | 0.2147 | PI3K-Akt signaling pathway | 0.5537 |
| FADS2 | Cellular lipid metabolic process  Lipid biosynthetic process | 0.0004  0.0007 | Linoleoyl-CoA desaturase activity  Acyl-CoA desaturase activity | 0.1189  0.3160 | alpha-Linolenic acid metabolism | 0.0514 |
|  |  |  |  |  | Fatty acid metabolism | 0.1271 |
|  |  |  |  |  | PPAR signaling pathway | 0.1562 |

Supplementary Table 11

Correlation coefficient of expression quantity between differential miRNAs and target genes, differential miRNAs and targeted regulated DELs

| MiRNA ID | DELs ID/ target genes | Pearson correlation | *P*-value |
| --- | --- | --- | --- |
| bta-miR-421 | TCONS_00104103 | -0.4558412 | 0.256317 |
| bta-miR-421 | TCONS_00082721 | -0.4602462 | 0.25116 |
| bta-miR-145 | TCONS_00082721 | -0.2439811 | 0.560365 |
| bta-miR-145 | TCONS_00054231 | -0.1671581 | 0.692368 |
| bta-miR-145 | TCONS_00027906 | -0.1810123 | 0.667943 |
| bta-miR-145 | TCONS_00045874 | -0.3571555 | 0.385103 |
| bta-miR-145 | TCONS_00083670 | -0.2404685 | 0.566201 |
| bta-miR-455-3p | TCONS_00054231 | -0.4809257 | 0.227658 |
| bta-miR-455-3p | TCONS_00083670 | -0.4718098 | 0.237873 |
| bta-miR-455-3p | TCONS_00184475 | -0.4302188 | 0.287348 |
| bta-miR-222 | TCONS_00184475 | -0.4288225 | 0.28909 |
| bta-miR-1343-3p | TCONS_00184475 | -0.6599821 | 0.074918 |
| bta-miR-1343-3p | TCONS_00119433 | -0.5662182 | 0.14343 |
| bta-miR-1343-3p | TCONS_00119434 | -0.7520586 | 0.031371 |
| bta-miR-1343-3p | TCONS_00045874 | -0.6081713 | 0.109661 |
| bta-miR-1343-3p | TCONS_00054231 | -0.6453585 | 0.083953 |
| bta-miR-1343-3p | TCONS_00004679 | -0.6005058 | 0.115452 |
| bta-miR-370 | TCONS_00175773 | -0.6855725 | 0.06054 |
| bta-miR-370 | TCONS_00147331 | -0.5255953 | 0.180962 |
| bta-miR-370 | TCONS_00143115 | -0.5036658 | 0.203184 |
| bta-miR-370 | TCONS_00143117 | -0.3344853 | 0.418048 |
| bta-miR-331-3p | TCONS_00027906 | -0.4076667 | 0.316091 |
| bta-miR-197 | TCONS_00027906 | -0.411413 | 0.311226 |
| bta-miR-197 | TCONS_00150126 | -0.6109198 | 0.107625 |
| bta-miR-197 | TCONS_00066920 | -0.552616 | 0.155469 |
| bta-miR-197 | TCONS_00054231 | -0.499543 | 0.207514 |
| bta-miR-2387 | TCONS_00083670 | -0.3837501 | 0.347989 |
| bta-miR-2387 | TCONS_00172817 | -0.5060242 | 0.200729 |
| bta-miR-2387 | TCONS_00091027 | -0.0601868 | 0.887422 |
| bta-miR-2387 | TCONS_00004679 | -0.3635594 | 0.376011 |
| bta-miR-370 | MTM1 | -0.67037 | 0.068865 |
| bta-miR-370 | ANO1 | -0.53606 | 0.170837 |
| bta-miR-370 | PLA2G15 | -0.16585 | 0.694692 |
| bta-miR-370 | FADS2 | -0.37496 | 0.360072 |
| bta-miR-2387 | SRRM2 | -0.38986 | 0.339708 |
| bta-miR-2387 | NDRG1 | -0.30245 | 0.46654 |
| bta-miR-2387 | PPP2R5A | -0.53811 | 0.168899 |
| bta-miR-455-3p | SMAD2 | -0.06563 | 0.877291 |
| bta-miR-455-3p | HADHB | -0.37852 | 0.355148 |
| bta-miR-1343-3p | RXRA | -0.32978 | 0.425027 |
| bta-miR-1343-3p | DIS3L2 | -0.52252 | 0.183994 |
| bta-miR-1343-3p | PLA2G4F | -0.58875 | 0.124665 |
| bta-miR-331-3p | PLTP | -0.53063 | 0.176051 |
| bta-miR-331-3p | SPHK2 | -0.23234 | 0.579784 |
| bta-miR-331-3p | LEP | -0.42272 | 0.296757 |
| bta-miR-331-3p | CYP26B1 | -0.56628 | 0.143376 |
| bta-miR-331-3p | TBL1X | -0.45372 | 0.258825 |
| bta-miR-197 | TBL1X | -0.34378 | 0.404404 |
